# Monte Carlo simulations of time-resolved blood flow index: times-of-flight beyond ∼1 ns are necessary for brain-dominated measurements

**DOI:** 10.1117/1.NPh.13.2.025003

**Published:** 2026-03-30

**Authors:** Dominic W. Hill, Anurag Behera, Octave Etard, Yuqian Zhang, Alexandra Tran-Van-Minh, Alexander Ruesch, Lior Ness, Chloe Maine, Amir Salehi Lashkajani, Veronika Parfentyeva, Navjit Singh, Alexander Antrobus, Rachel Prudden, Pablo Villar Sanjurjo, Stella Avtzi, Tanja Dragojević, Dawid Borycki, Matthew T. Valley, Robert J. Cooper

**Affiliations:** a CoMind Technologies, London, United Kingdom; b UCL, Department of Medical Physics and Biomedical Engineering, Biomedical Optics Research Laboratory, London, United Kingdom

**Keywords:** Monte Carlo simulation, cerebral blood flow, time-resolved, diffuse correlation spectroscopy, speckle contrast optical spectroscopy

## Abstract

**Significance:**

Achieving adequate brain sensitivity remains a significant obstacle to noninvasive optical measurements of pulsatile cerebral blood flow. Increasing source-detector separation (SDS), utilizing time-of-flight (ToF) information, and/or increasing acquisition frequency can increase brain sensitivity. However, optimizing these parameters is nontrivial and must balance the need to achieve sufficient signal-to-noise ratio.

**Aim:**

We aim to guide hardware optimization by evaluating the benefits of ToF gating, autocorrelation time-lag gating, and increased source-detector separation in recovering pulsatile cerebral blood flow.

**Approach:**

Monte Carlo simulations of ToF-resolved pulsatile blood flow index at 1064 nm were performed. A simulation and analysis pipeline is presented and validated against phantom and *in vivo* measurements.

**Results:**

Brain sensitivity deteriorates for autocorrelation time-lags >1 to 10  μs. Continuous-wave diffuse correlation spectroscopy (CW-DCS) at 40 mm SDS achieves equivalent brain sensitivity to time-resolved measurements with a 1.2 ns ToF gate. For median adult brain depths, a 31 mm SDS (CW-DCS), 35 mm SDS (speckle contrast optical spectroscopy), or 0.9 ns ToF are needed for brain-dominated signals. For the 85th percentile of brain depth, these thresholds increase to 40 mm, 46 mm, and 1.2 ns, respectively.

**Conclusions:**

We quantitatively compare source-detector separation, ToF gating, and acquisition frequency and identify crucial performance requirements for clinically viable optical monitors of cerebral blood flow.

## 1 Introduction

The development of a technology that can reliably and noninvasively measure human cerebral blood flow (CBF) has been a fundamental goal of biomedical optics research for three decades.^1^^–^^3^ In that time, a variety of methods have been proposed to achieve this target based on the transmission of coherent light through the tissues of the head and the examination of the dynamics of the light field that emerges. Foremost among these methods is continuous-wave diffuse correlation spectroscopy (CW-DCS).^1^^,^^4^^–^^7^ As coherent light propagates through tissue, it undergoes multiple scattering events, accumulating phase-shifts along every optical path. The interference of light traversing different paths generates a speckle pattern at the detector. As scatterers in the medium move, these phase-shifts change, leading to temporal fluctuations in speckle intensity.^8^ In CW-DCS, the movement of these scatterers (primarily red blood cells) can be quantified via the temporal autocorrelation of the fluctuating speckle intensity, which takes a form proportional to $\exp(-\alpha D_{B}\tau )$,^8^^–^^11^ where $\alpha D_{B}$ represents the scatterer Brownian diffusion coefficient and τ is the autocorrelation lag time. It is well established that changes in $\alpha D_{B}$ in a given tissue are directly and linearly related to blood flow in that tissue.^4^^,^^12^^,^^13^ As a result, $\alpha D_{B}$ (or derived parameters proportional to $\alpha D_{B}$) are invariably referred to as a “blood flow index” (BFi).

CW-DCS has been successfully applied in a vast range of laboratory and clinical research environments.^1^^,^^14^ In recent years, numerous related techniques have emerged that build on the foundation CW-DCS has provided. This includes techniques like speckle contrast optical spectroscopy (SCOS)^15^ that seek to overcome the signal-to-noise challenges of CW-DCS through multi-pixel detection and by replacing fast autocorrelation measures with spatial speckle contrast approaches. Recent work has demonstrated the superiority of SCOS methods over CW-DCS methods in terms of CBF contrast to noise when pixel counts are sufficiently high.^16^ However, SCOS is typically employed using the relatively slow acquisition rates associated with typical camera sensors, which can make it challenging to capture the fast speckle dynamics associated with blood flow, particularly in the brain.

One of the most exciting technological trends of recent years has been the development of methods that combine sensitivity to speckle dynamics with photon time-of-flight (ToF) information. These methods, which include time-domain DCS (TD-DCS)^17^^–^^19^ and time-resolved interferometric approaches,^20^ have the potential to provide an exquisitely powerful optical neuromonitoring technology that can yield measurements of ToF-resolved BFi as well as absolute measurements of the optical characteristics of tissue.

However, despite these many significant developments across a range of technologies, no optical measurement of CBF has yet been successfully translated into a medical device, nor directly impacted patient care.^21^

A fundamental challenge in the development of an effective and clinically relevant device for speckle-based determination of human CBF is the necessity of resolving individual speckles, which (given that most collected light will have traversed many centimeters of tissue), will inherently exhibit low optical intensities. This challenge is compounded by the desire to measure CBF at high sample rates, well above the pulse rates observed in patients. Measuring at above pulsatile rates is critical because (a) the pulse waveform is increasingly believed to contain valuable information related to neurophysiological state^22^^–^^26^ and (b) high sample rates negate the risk of aliasing high frequency physiological changes into lower frequency bands. The latter is vital to ensuring robust measurements of CBF changes over longer, yet highly clinically relevant, timescales. The measurement of pulsatile CBF is challenging not only because it requires high signal-to-noise and high sampling rates but also because the pulsatile CBF signal is masked by pulsatile blood flow in the superficial layers.^7^^,^^27^^,^^28^ Both layers exhibit pulsatile blood flow waveforms, and although these waveforms can and will differ in amplitude, phase, and features, they will also overlap in time and frequency. Disentangling the superficial and cerebral pulsatile BFi waveforms is therefore a very significant engineering challenge, and one that has yet to be satisfactorily addressed by the biomedical optics research community.

The key to overcoming this challenge is to ensure that any measurement system has sufficient brain sensitivity and selectivity. Herein, we define brain sensitivity as the ratio of the sensitivity of the recovered signal to changes in the brain to the total sensitivity of the recovered signal to changes across the entire measured volume. The brain sensitivity of CW-DCS and related methods is known to be significantly higher than that of continuous wave near infrared spectroscopy (CW-NIRS) methods, which seek to measure cerebral hemoglobin concentrations.^28^^,^^29^ At a source–detector separation (SDS) of 3 cm, CW-DCS is ∼3 times more sensitive to changes in the brain than CW-NIRS, and this advantage only increases for longer SDS.^28^^,^^29^ This is because speckle-based methods are sensitive to the movement of optical scatterers in tissue, and the greater the number of scattering events the collected light has undertaken, the greater the impact on the rate of speckle decorrelation. Because the number of scattering events and optical path length are approximately proportional,^30^ CW-DCS and related approaches are intrinsically more sensitive to light that has travelled (on average) deeper into the tissue.

For technologies that rely on speckle dynamics to infer CBF, brain sensitivity can be improved via three fundamental mechanisms: increasing SDS,^31^^,^^32^ increasing acquisition rate,^33^ or utilizing ToF information.^34^^,^^35^

Increasing SDS and employing ToF information enhance brain sensitivity by increasing the proportion of detected photons that have traversed a deeper path into tissue.^34^ As SDS increases so does the average pathlength of detected photons and, therefore, the proportion of detected photons that have reached the brain. However, both the total number of detected photons and the number of detected photons that have reached the brain exponentially decrease with SDS. In time-resolved methods, photons traversing longer pathlengths can be explicitly selected using appropriate ToF gating approaches. Time-resolved methods also have the significant advantage that a short (or even null) SDS can be used to maximize the number of detected photons that have travelled deep into tissue while still having the potential to filter out photons that travel shorter paths.^35^ A further advantage is that time-resolved methods allow separate measurements of both superficial and deep BFi using a single source-detector configuration.

Increasing the speed at which a speckle field is sampled, i.e., increasing the instrument acquisition rate, can also provide improved brain sensitivity.^28^^,^^33^ The temporal fluctuation rate of the detected light is proportional to both the number of scattering events the collected photons have undertaken and the diffusion rate of the dynamic scatterers. The blood flow in the densely vascularized brain tissue is ∼3 to 10 times higher than that of the scalp.^36^^–^^39^ Increasing the acquisition rate and (for example) examining only the shorter autocorrelation lags that a faster acquisition rate provides can therefore act like a frequency filter, wherein the recovered signal can be preferentially biased to contain more of the fast brain-dominated signal relative to the slower, superficial-dominated signals.

With a hard limit on photon budget set by the maximum permissible exposures specified in ISO:60825, there are clearly instrument design tradeoffs between increasing SDS, increasing the maximum measurable ToF, and/or increasing acquisition rate. It is well understood that increasing any of these three parameters in any instrument will reduce photon count per sample. This paper expands upon previous research by concurrently investigating the comparative advantages of these three methods.^14^^,^^28^^,^^31^^,^^34^

Several prior studies have simulated TD-DCS from first principles, including the works by Cheng et al.,^40^ Huang et al.,^41^ Colombo et al.,^42^ and Mazumder et al.^43^ These studies investigated the fundamental mechanisms of photon dynamics in TD-DCS, the impact of instrument response functions (IRFs) and gating width, and strategies for optimizing system parameters to enhance brain flow sensitivity. However, as noted, for instance, by Cheng et al., realistic conditions under which TD-DCS clearly outperforms CW-DCS could not be identified, largely due to the broad IRFs and limited timing precision characteristics of current TD-DCS systems.^17^^,^^19^^,^^42^ The advent of interferometric methods permits time-resolved measures with significantly narrower IRFs and a long laser coherence length,^44^^,^^45^ which remains under-explored in simulation. Furthermore, the aforementioned simulation studies focused on brain sensitivity or selectivity for fixed brain and scalp flow. Here, we expand on prior work to investigate brain sensitivity when the TD-device timing precision is not a limiting factor and examine how device characteristics can impact the recovery of the pulsatile CBF waveform, a key signal in clinical settings.

In this paper, we describe the design and implementation of a multi-layer, ToF resolved Monte Carlo simulation of dynamic blood flow to investigate and quantify the relative benefits of “ToF gating,” ”SDS gating,” and “lag gating” for maximizing the brain sensitivity of BFi measurements. The aim of this simulation stack is to support the design of instrumentation to optimize the performance of speckle-based measurements of human CBF. The outputs of this simulation work have guided the development of a novel time-resolved interferometric optic neuromonitoring technology that is described in a companion paper submitted concurrently with this work.

## 2 Methods

**Fig. 1:**
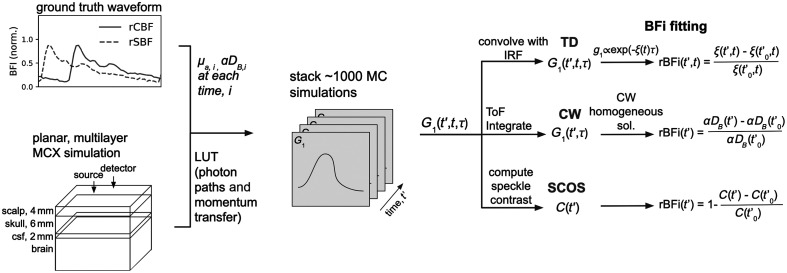
Schematic showing the simulation and subsequent BFi fitting of a pulsatile time series. A look up table of photon partial pathlengths and momentum transfers is first generated using MCX. Ground truth $\alpha D_{B,\text{scalp}}$, $\alpha D_{B,\text{brain}}$, $\mu_{a,\text{scalp}}$, and $\mu_{a,\text{brain}}$ for the time series are produced by modifying an *in vivo-*derived arterial blood pressure waveform. ToF resolved $G_{1}$ is computed at each point in the time series and stacked along the time dimension to mimic a time resolved measurement. The $G_{1}$ is then either, convolved with an IRF, integrated along the ToF axis or used to compute speckle contrast for TD, CW, and SCOS measurements respectively. Finally, rBFi is extracted by fitting the time series using the appropriate BFi fitting method and calculating the relative difference from the initial time point.

We established multi-layer Monte Carlo simulations of measurements of the human head with appropriate adult-equivalent tissue thicknesses and pulsating brain and scalp compartments (Fig. 1). The Monte Carlo package MCX^46^ was used to generate a lookup table of photon weights, partial pathlengths, and momentum transfer for the chosen geometry. From this lookup table, the sensitivity of different SDS, ToFs, and lags are evaluated. To simulate measurements of pulsatile blood flow, temporal autocorrelations are computed for each point in a ground truth time series and then stacked along the time axis. This methodology is described in further detail in Sec. 2.6.

### 2.1 Simulation Setup

Two simulation geometries were used: a multilayer planar geometry and an anatomical head model.

The multilayer slab configuration consists of four layers designed to approximate the scalp, skull, cerebrospinal fluid (CSF), and brain. Table 1 displays the default simulated optical properties at a wavelength of 1064 nm. This wavelength was chosen because the use of 1064 nm light presents significant advantages over traditional NIR wavelengths for measurements of BFi, due to the lower tissue reduced scattering coefficients, lower tissue absorption coefficients, and higher maximum permissible exposure (MPE).^47^ The best-in-class devices now typically operate at 1064 nm.^44^^,^^48^^–^^50^ Table 1 also presents the default thicknesses of the different layers of the multi-layer model. These values were collated from a range of anatomical resources.^36^^,^^48^^,^^51^^–^^53^ In addition to the median case, a range of skull thicknesses was also simulated based on the distribution over the pre-frontal cortex over the adult population.^36^^,^^52^ The distribution of skull and distance-to-brain thicknesses extracted from the literature is presented in Fig. S1 in the Supplementary Material.

**Table 1:** Default simulation optical properties and layer thicknesses.

	$\mu_{a}$ (${\mathrm{mm}}^{-1}$)	$\mu_{s}^{\prime }$ (${\mathrm{mm}}^{-1}$)	G	n	BFi baseline (${\mathrm{mm}}^{2}\;s^{-1}$)	Thickness (mm)
Scalp	∼0.012	0.84	0.9	1.4	$1\times 10^{-6}$ (range $0.5\times 10^{-6}\text{–}1.7\times 10^{-6}$)	4
Skull	0.013	0.84	0.9	1.4	$2\times 10^{-8}$	6 (range 3–11)
CSF	0.012	0.009	0.9	1.33	$5\times 10^{-8}$	2
Brain	∼0.017	0.84	0.9	1.4	$5\times 10^{-6}$ (range $4\times 10^{-6}\text{–}15\times 10^{-6}$)	—

A single example anatomical head model was chosen at random from the ScatterBrains dataset^53^ (subject 3), with a simulated probe placed on the pre-frontal lobe. The scalp-to-brain distance at the sensors’ barycenter was 10 mm for all simulations. This distance varied between 10 and 14 mm across the different probe locations due to natural physiological variation. For reference, the mean adult distance-to-brain is 12 mm (Fig. S1 in the Supplementary Material) for the pre-frontal lobe. The subject was a 24-year-old White Hispanic/Latino female.^53^ The optical properties used are detailed in Table 1.

The simulated scalp $\alpha D_{B}$ was varied over a range $(0.5\times 10^{-6}\text{ to }1.7\times 10^{-6})\;{\mathrm{mm}}^{2}\;s^{-1}$ and cerebral $\alpha D_{B}$ from $(4\times 10^{-6}\text{ to }15\times 10^{-6})\;{\mathrm{mm}}^{2}\;s^{-1}$. This range was selected to match the observed *in vivo* $g_{1}$ decorrelation rates at early and late ToF for a cohort of 25 healthy adult subjects used as part of the benchmarking of our simulations which is described in Sec. 3.2. This range is also consistent with values used in the literature.^36^^,^^38^^,^^48^^,^^54^

The sensitivity of the simulation results to the choice of optical properties is detailed in Fig. S2 in the Supplementary Material.

Using the outputs of our MCX simulations, the ToF resolved temporal field autocorrelation function ($G_{1}$, or $g_{1}$ when normalized by the first lag) was computed for the relevant layer-wise absorption and blood flow indices using the following Eqs. (4) and (5):

$$G_{1}(t_{s},\tau_{d})=\frac{1}{N_{p}}\overset{N_{p}}{\underset{p=1}{\sum }}w_{p}(t_{s})\;\exp(-\frac{1}{3}\overset{N_{m}}{\underset{n=0}{\sum }}\;Y_{p,n}(t_{s})k_{0}^{2}\;\langle \Delta r_{n}^{2}(\tau_{d})\rangle ),$$ (1)

where $w_{p}(t_{s})$ represents the attenuation of the light due to absorption from the turbid the medium such that

$$w_{p}(t_{s})=\exp(-\overset{N_{m}}{\underset{n}{\sum }}\mu_{a,n}\;L_{p,n}(t_{s})).$$

Here, $t_{s}$ is the photon ToF and $\tau_{d}$ is the autocorrelation lag time/decorrelation time. The term $Y_{p,n}(t_{s})=\sum q_{n}^{2}/2k_{0}^{2}$ is the total dimensionless momentum transfer of photon, p, through layer n, where the momentum change of a photon in a given scattering event is given by ${\overset{\to }{q}}_{n}={\overset{\to }{k}}_{\text{out}}-{\overset{\to }{k}}_{\text{in}}$, where ${\overset{\to }{k}}_{\text{in}}\;$ and ${\overset{\to }{k}}_{\text{out}}$ are the photon wavevectors before and after a scattering event in layer n and $k_{0}=|\overset{\to }{k_{\text{in}}}|=|\overset{\to }{k_{\text{out}}}|$. The term $N_{p}$ is the number of detected photons, $N_{m}$ is the number of tissue types the photons have travelled through in the medium, $\mu_{a,n}$ is the absorption coefficient in layer n, and $L_{p,n}(t)$ is the partial pathlength of the p’th photon through layer n. It is assumed that the dynamic scatterers are undergoing Brownian diffusion^4^^,^^8^^–^^11^ such that the mean squared displacement $\langle \Delta r_{n}^{2}\rangle =6\alpha D_{B,n}\tau_{d}$.

Measurements by a CW-DCS-like device are simulated by integrating the ToF resolved $G_{1}$ [Eq. (1)] over photon ToF. Assuming a known coherence factor, β, the simulated normalized electric field autocorrelation function can then be converted directly to $g_{2}$ through the Siegert relation^55^

$$g_{2}(\tau_{d})=1+\beta |g_{1}(\tau_{d}){|}^{2}.$$ (2)

To simulate a SCOS-like measurement, the simulated $G_{1}$ is converted into the squared speckle contrast, C, through the standard formula^15^

$$C=K(T{)}^{2}=\frac{2\beta }{T}\int_{0}^{T}(1-\frac{\tau_{d}}{T})|g_{1}(\tau_{d}){|}^{2}d\tau_{d},$$ (3)

where T is the exposure time.

### 2.2 Sensitivity Metrics

The sensitivity of the measured $g_{1}$ to a percentage change in flow in layer n is given by the following expression:

$$s_{n}^{\prime }(t,\tau )=\alpha D_{B,n}\partial_{\alpha D_{B,n}}g_{1}(t,\tau ),$$ (4)

where $\alpha D_{B,n}$ is the baseline diffusion coefficient in layer n. In hardware, $g_{1}$ is sampled discretely along the τ axis at the acquisition frequency, $f_{\mathrm{acq}}=1/\Delta \tau$. The total sensitivity to a given layer for a given device acquisition frequency is defined as the integral from the minimum resolvable lag for the given acquisition frequency,$\tau_{\min}=1/f_{\mathrm{acq}}$, upwards

$$S_{n}(t,\tau_{\min})=\int_{\tau_{\min}}^{\infty }\alpha D_{B,n}\partial_{\alpha D_{B,n}}g_{1}(t,\tau^{\prime })d\tau^{\prime }.$$ (5)

In practice, an integration upper bound of 1 s is used here, as the sensitivity decays to near zero long before this limit in all cases simulated. $\partial_{\alpha D_{B,n}}g_{1}(t,\tau_{j})$ may be directly computed from a Monte Carlo simulation by inserting the value for $G_{1}$ in Eq. (1), normalizing by the first lag, and computing the derivative with respect to the value $\alpha D_{B,n}$ in layer n, this is outlined in further detail in Supplementary Material Sec. 1. The integral in Eq. (5) is evaluated numerically using Simpson’s rule, Python function scipy.integrate.simpson.

Note that the sensitivity to relative rather than absolute $\alpha D_{B}$ changes are calculated here. We consider this a more valuable metric because baseline flow values in different tissues can vary by several orders of magnitude, whereas the pulsatility index (the pulse amplitude divided by its mean) tends to be more similar between tissues.^29^^,^^56^ Furthermore, we have deliberately chosen a brain sensitivity metric that is independent of the BFi fitting method. The brain sensitivity of a given modality is then ratio of this value evaluated for the both the brain and the scalp, i.e.,

$$\text{Brain sensitivity}=\frac{S_{\mathrm{CBF}}}{S_{\mathrm{CBF}}+S_{\mathrm{SBF}}}.$$ (6)

### 2.3 Experimental Validation of the Simulation Stack

Simulations of ToF-resolved $G_{1}$ were validated against both phantom and *in vivo* measurements obtained with a prototype, time-resolved interferometric optical sensing device (CoMind R1), operating at 1064 nm. This device was recently presented and is described and extensively validated in a companion paper submitted concurrently with this work.^57^^,^^58^

To simulate ToF-resolved measurements, all simulated $G_{1}s$ were convolved along the ToF axis with the CoMind R1 device instrument response function (IRF), which has a full-width at half-maximum of ∼125  ps, and then resampled from their linear lag-axis to a logarithmic scale,^59^ to match the hardware post-processing. The IRF applied is displayed in Fig. S5 of the Supplementary Material. The effects of the width of the IRF on the predicted depth sensitivity are also presented in Fig. S4 in the Supplementary Material.

#### 2.3.1 *In vitro* benchmarking

Measurements on bilayer intralipid phantoms were used to validate that the depth selectivity of the simulation matched those derived from experimental data. A more complete description of the CoMind R1 device and associated phantoms is presented elsewhere,^57^^,^^58^ but in brief, the upper layer of the phantom was 10 mm deep, with a 75 μm layer of Mylar used to separate the upper from the lower intralipid layers. As a means to mimic a two-layer system with a higher $\alpha D_{B}$ in the lower layer than in the upper (analogous to brain and scalp *in vivo*), the reduced scattering coefficient in the lower layer was modified by changing the intralipid concentration (intralipid volume fractions 0.04, 0.07, 0.14, and 0.21 corresponding to fitted reduced scattering coefficients of 0.55, 1.0, 1.8, and $2.6\;{\mathrm{mm}}^{-1}$, respectively). In all bilayer measurements, the upper layer reduced scattering was $\mu_{s}^{\prime }=0.55\;{\mathrm{mm}}^{-1}$. As a control, a homogeneous volume of intralipid solution with the same concentrations was also measured. Each phantom configuration was measured for 110 s at a sample rate of 47.7 Hz. BFi fitting was performed for each point in this time series and the mean and standard deviation calculated. No absorber was used in any of these phantom configurations and the solution is assumed to have an equivalent absorption coefficient to water, $\mu_{a}=0.015\;{\mathrm{mm}}^{-1}$.^60^ In the simulation, the refractive index was set to 1.33 and an anisotropy factor g=0.53 was used, which is appropriate for intralipid at 1064 nm.^61^^,^^62^

#### 2.3.2 *In vivo* benchmarking

To ensure simulations were representative of *in vivo* measurements, a “scalp-occlusion” paradigm^17^^,^^63^ was used to obtain data with both minimal scalp blood flow and with unperturbed scalp blood flow in 25 healthy adult subjects. To achieve this, 25 healthy volunteers were recruited. All *in vivo* studies were conducted at CoMind’s laboratories in London, UK. The experimental protocol was approved by an in-house ethical review process that incorporated an independent external expert review. Prior written informed consent was obtained from each volunteer.

**Fig. 2:**
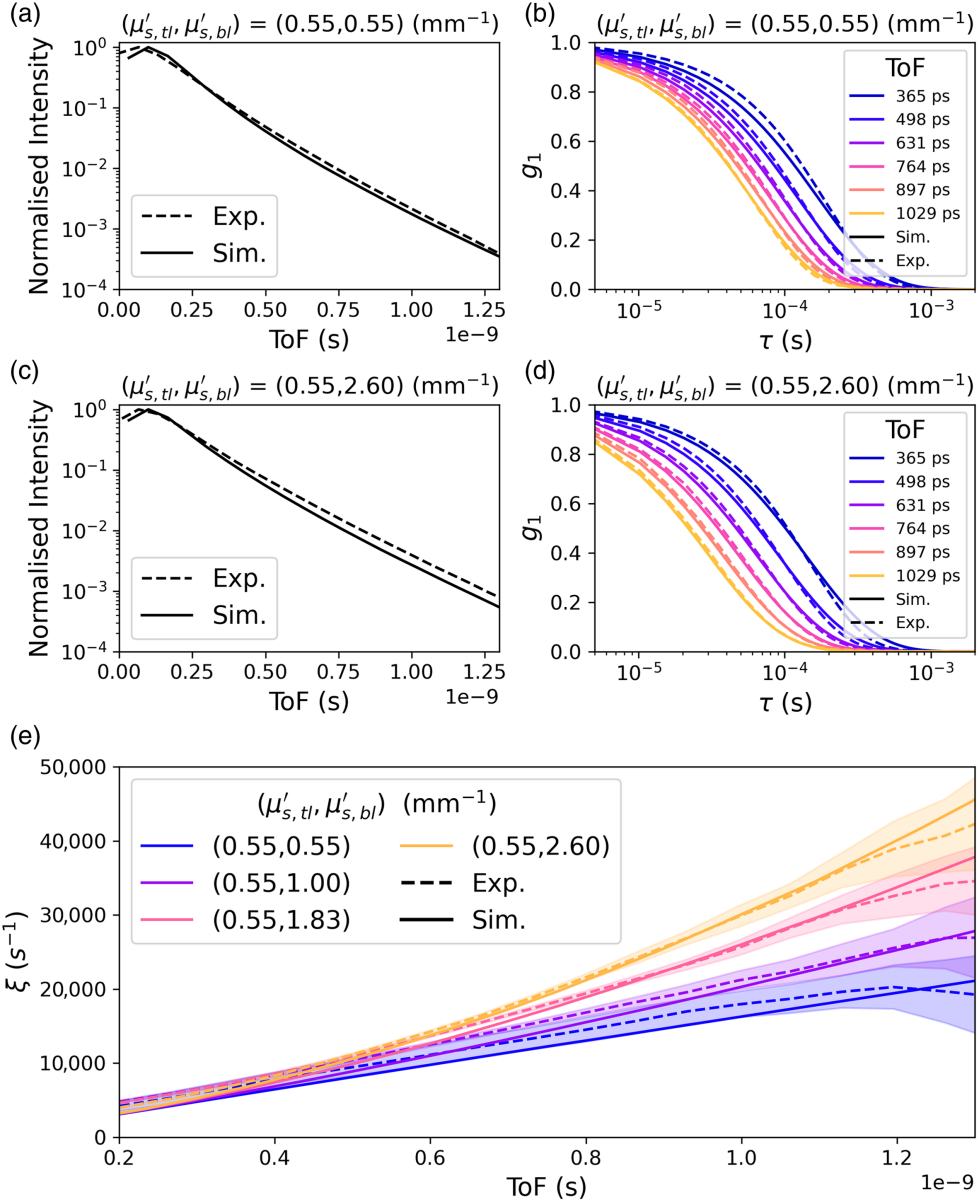
Upper two rows present example TPSFs (left) and $g_{1}s$ (right) for simulation and experiment from reference homogeneous and bilayer intralipid phantom measurements with different reduced scattering coefficients. In panel (e), the fitted decay coefficient ξ versus ToF for simulations and experiment are presented. In all figures, the solid line represents the result of the simulation and the dashed lines present the experimental benchmark. Experimental results are averaged over a 110 s measurement interval with shaded region in (e) representing the standard deviation in fitted ξ over this time. A single exponential fit is used for both simulation and experimental data.

The CoMind R1 optical sensor provides a single 10 mm SDS channel. The sensor was applied to the forehead at approximately FP1 in the EEG 10–20 system, with the midpoint between the source and detector aligned with the center of the subject’s eye socket, whereas the lower edge of the sensor was positioned ∼2  cm above the eyebrow line. Above the sensor, a wrap-around headband with an inflatable pressure cuff was applied in the configuration shown in Fig. 2. First, a 3 min baseline recording was undertaken. The pressure cuff was then inflated to 120 mmHg. As the pressure increases in the cuff, the strips above and below the probe tighten and occlude the scalp blood flow in the region of the sensor, without perturbing the sensor itself. Another 3 min of data was recorded in this occluded state. The pressure was then released, and 3 further minutes of data were collected as the scalp blood flow returned to the baseline state. One trial was performed per subject. The cohort consisted of 16 males subjects and nine females, the mean age was 35±9 years, and the mean Monk skin tone was 2.2±1.7.

Optical property fits, using a Monte Carlo fitting method, were performed on the TPSFs to extract the variance in reduced scattering coefficients across the 25 subjects. Meanwhile, the distribution of decorrelation rates was extracted using single-exponential fitting at early and late ToFs (lags below a threshold of $g_{1}=0.5$ were rejected in line with the fitting approaches applied to our simulated data and described above). Together, these data were used to ensure that the ranges of simulated $\alpha D_{B,\text{scalp}}$, $\alpha D_{B,\text{brain}}$, $\mu_{s}^{\prime }$ and decorrelation rates were representative of those seen *in vivo*.

### 2.4 Simulation of Spatial Distributions of Sensitivity in an Anatomical Head Model

To provide a comparison to our multilayer slab simulation and to better relate our brain sensitivity results to human anatomy, simulated spatial sensitivity distributions were computed that map the sensitivity to changes in $\alpha D_{B}$ in the selected anatomical head model.

Four SDS were simulated: 10 mm (for both CW and TD cases), then 20, 30, and 40 mm for CW only cases. The barycenter of the source-detector pair was maintained at approximately the same position (within 1 mm) for all four simulated separations. For each MCX simulation, $4\times 10^{9}\text{ photons}$ were launched. Due to the substantial memory requirements for storing photon momentum transfer and partial path lengths for each $0.5\;{\mathrm{mm}}^{3}$ voxel, ∼500 trials of the same simulation were executed for each source-detector configuration. Each trial utilized the same Monte Carlo random number seed, with parameters saved for only a subset of voxels. These subsets were then stitched together to reconstruct the full image, effectively yielding a sensitivity map equivalent to a single simulation with $4\times 10^{9}\text{ photons}$. To improve photon statistics, the computed sensitivity was averaged over three voxels in the out of plane dimension to make the final voxel resolution of the image $0.5\times 0.5\times 1.5\;{\mathrm{mm}}^{3}$.

### 2.5 Simulation of Pulsatile Time Series

Pulsatile $\alpha D_{B}$ time series were simulated for a variety of different layer thicknesses and optical properties in our multi-layer slab model. A schematic of this simulation pipeline is presented in Fig. 1. In each case, the pulsatility index (the pulse amplitude divided by the mean BFi value) was set to be 0.8 in both the scalp and the brain, consistent with the values found in Urner et al.^56^ To create a time series, 1000 Monte Carlo simulations, each with optical properties corresponding to a different measurement time point, were stacked. $4\times 10^{9}\text{ photons}$ were used to generate the lookup table containing the photon path lengths and momentum transfers. A pulsatile waveform derived from an *in vivo* arterial blood pressure measurement, sampled at 50 Hz, was used as the basis for this simulation. The same waveform was used as the basis for both simulated CBF and simulated scalp blood flow (SBF), but (in the example presented here) phase-shifted by π/4. This phase shift can be arbitrarily adjusted to match physiologically observed phase shifts. Here, we use π/4 for convenience, but in practice, it could be smaller. The effect of changing this phase shift is presented in Fig. S3 in the Supplementary Material. To simulate simultaneous changes in blood volume, absorption was also modulated (2% to 4% from baseline) in the brain and scalp compartments. The absorption waveform was selected as a downsampled, low-pass filtered version of the flow waveforms to mimic phenomenological similarity with recovery using typical CW-NIRS measurements.^64^

### 2.6 Recovery of Pulsatile BFi

Recovery of BFi (in the form of $\alpha D_{B}$ in units of ${\mathrm{mm}}^{2}/s$) for simulated CW-DCS-like measurements was performed using the homogeneous solution to the correlation diffusion equation.^65^ In the case of simulated SCOS measurements, the speckle contrast was used directly as a measurement of BFi.

In a similar manner, for ToF-resolved measurements, the time domain solution to the correlation diffusion equation in a homogeneous medium was used. This consists of a single exponential function that is applied per-ToF as a 1-parameter fit:

$$g_{1}(t_{s},\tau_{d})=e^{-\xi (t_{s})\tau_{d}}$$ (7)

where $\xi (t_{s})$ is the decay coefficient (or decay rate, in units of kHz), $t_{s}$ is the photon ToF, and $\tau_{d}$ is the $g_{1}$ “lag.” Here, $\xi (t_{s})$ can be related to the BFi (Brownian diffusion coefficient of the dynamic scatterers, $\alpha D_{B}$) via the Eq. (8):

$$\xi (t_{s})=2k^{2}\mu_{s}^{\prime }(\frac{c}{n})\alpha D_{B}t_{s}.$$ (8)

where k is the light wavenumber, $\mu_{s}^{\prime }$ is the reduced scattering coefficient, c is the speed of light in a vacuum, and n is the refractive index of the medium. Prior to fitting, $g_{1}$ points below the minimum threshold of $g_{1}=0.5$ are discarded, provided that a minimum of six lag points remain for the fitting process. However, if $g_{1}$ decays below 0.5 within the initial six lag points, these first six points are utilized instead.

### 2.7 Evaluation of Pulsatile CBF Recovery

Once BFi is extracted to yield a pulsatile time series in each simulation, the result is evaluated based on the correlation of the relative changes in BFi (rBFi) to the ground truth waveforms simulated in the brain and scalp layers (simulated rCBF and rSBF, respectively).^27^ In each case, rBFi is determined relative to the first time point in the simulated time-series. Note that in the case of simulated SCOS measures, the relative contrast is subtracted from 1 so that the recovered rBFI is positively correlated with flow.

The Pearson correlation-coefficient is taken between each recovered rBFi and the simulated rCBF waveform, and between each rBFi and the simulated rSBF to quantify the similarity between the recovered pulsatile waveform and the pulsatile waveforms of the brain and scalp layers. Note that root mean-squared error was also tested as a metric; however, it yielded identical results to Pearson’s R, so only the latter is presented here.

## 3 Results

An overview of the results sub sections is provided below.

• **Section 3.1 *In vitro* benchmarking:** In this section, we validate our ability to simulate sensitivity to flow at depth in two-layer phantoms. Reduced scattering coefficient is varied in the different phantom layers to mimic the higher physiological decorrelation rates in the brain than the scalp.
• **Section 3.2 *In vivo* benchmarking**: In this section, we validate that our simulations are representative of *in vivo* measurements by comparing them with data collected from 25 subjects under baseline and scalp-occlusion conditions. Using a multilayer planar geometry simulation, we determined the range of $\alpha D_{B,\text{scalp}}$ and $\alpha D_{B,\text{brain}}$ that yields $g_{1}s$ consistent with the experimentally observed *in vivo* variation.
• **Section 3.3 Effects of ToF, SDS, and acquisition frequency:** In this section, a quantitative analysis of brain sensitivity as a function of source–detector separation, ToF, and autocorrelation lag is performed using planar multi-layer simulations. Brain sensitivity is assessed over the physiologically representative range of $\alpha D_{B,\text{scalp}}$ and $\alpha D_{B,\text{brain}}$ found in Sec. 3.2.
• **Section 3.4 Anatomical model visualization:** This section focuses on the spatial mapping of flow sensitivity in a realistic head geometry, using an anatomical head atlas simulation.
• **Section 3.5 Pulsatile BFi recovery:** This section evaluates the recovered pulsatile blood-flow index (BFi) waveforms, which more closely represents the clinical neuromonitoring target, for different SDS, ToF gates, and device hardware, using a multilayer planar geometry simulation.
• **Section 3.6 Influence of brain depth:** This section assesses how population variability in distance-to-brain affects the brain sensitivity thresholds for recovery of pulsatile BFi established in Sec. 3.5. The assessment was performed by varying distance-to-brain, within multilayer planar geometry simulations.

### 3.1 *In Vitro* Benchmarking

This section presents results for the comparison of Monte Carlo simulations against *in vitro* phantom measurements. The aim of this investigation was to validate the simulated sensitivity to depth resolved flow by examining agreement between simulations and experimental measurements of homogeneous and bilayer intralipid phantoms, as described in Sec. 2.3.1. Figure 2 displays simulated temporal point spread functions [TPSFs, Figs. 2(a) and 2(c)] and $g_{1}s$, (b) and (d), alongside the experimental measurements for the homogeneous and bilayer measurements. Agreement between simulations and experiment is generally excellent for both TPSFs and $g_{1}s$, with the slight exception at very early ToF<100  ps, where a small mismatch in the TPSFs can be observed.

Figure 2(e) displays a comparison of BFi fits to simulations (solid lines) and experimental measurements (dashed lines and shaded regions representing the mean and standard deviations over the 110 s measurement window) of the bilayer phantoms and the homogeneous reference case. For the homogeneous phantom measurement, ξ is linearly proportional to ToF, as expected from diffusing-wave spectroscopy theory, Eq. (8). For the bilayer cases, ξ initially increases linearly with ToF at the same rate as the equivalent homogeneous case, but at later ToF its gradient, $d\xi /{\mathrm{dt}}_{s}$, increases. As reduced scattering coefficient in the bottom layer is increased, the change in gradient becomes more pronounced and the transition is observable earlier in ToF. This pattern is evident in both the experimental and simulated results, which are highly consistent with one another.

### 3.2 *In Vivo* Benchmarking

**Fig. 3:**
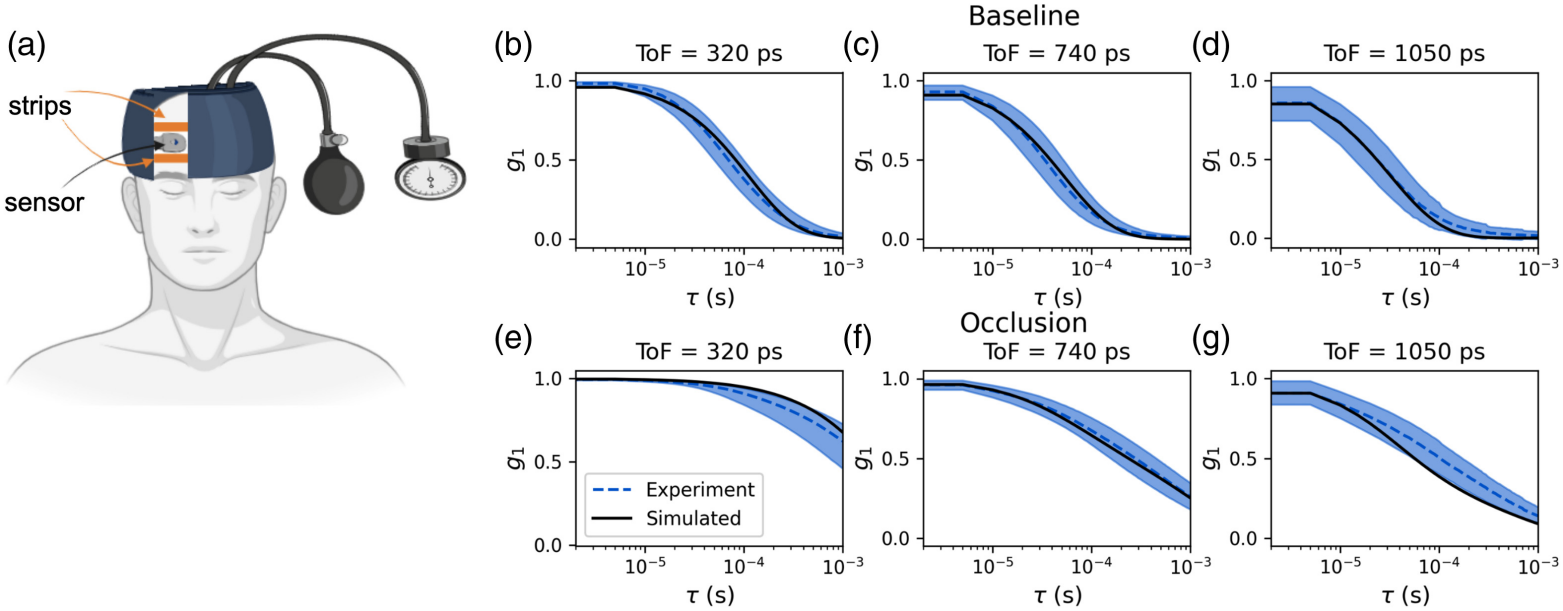
(a) A schematic of the scalp occlusion experiment. (b–d) Display simulated $g_{1}s$ (black) at different ToFs, for the baseline cases (no occlusion) alongside the measured $g_{1}s$ in the 25 subject dataset (solid blue line representing the median with the shaded regions indicating the upper and lower quartiles). (e–g) Show the equivalent simulated and experimental $g_{1}s$ for the N=25 measurements during application of the scalp occlusion.

This section presents the results of our *in vivo* benchmarking. Our primary goals were to (1) confirm that our simulations accurately represent *in vivo* measurements and (2) determine the range of simulated $\alpha D_{B,\text{scalp}}$ and $\alpha D_{B,\text{brain}}$ that aligns with the person-to-person variance observed in *in vivo* measurements, as detailed in Sec. 2.3.2. Simulations were compared with *in vivo* data taken from 25 adult subjects measured as part of a scalp occlusion study. Figure 3 shows simulated $g_{1}s$ at different ToFs alongside the *in vivo* median and interquartile range of $g_{1}$ for baseline (non-occluded) and occluded states. The simulated $g_{1}s$ are highly consistent with the *in vivo* data. The $\alpha D_{B}$ values needed to achieve these simulation results were an $\alpha D_{B,\text{brain}}$ of $4.8\times 10^{-6}\;{\mathrm{mm}}^{2}\;s^{-1}$ and an $\alpha D_{B,\text{scalp}}$ of $1.5\times 10^{-6}\;{\mathrm{mm}}^{2}\;s^{-1}$ for baseline and $\alpha D_{B,\text{scalp}}$ of $5\times 10^{-8}\;{\mathrm{mm}}^{2}\;s^{-1}$ during occlusion.

Optical property fitting of the temporal point spread function $\left(G_{1}(\tau =0)\right)$ was performed to find the best fit reduced scattering coefficient using a white Monte Carlo method.^66^ The best fit reduced scattering coefficient value was $(0.9\pm 0.3)\;{\mathrm{mm}}^{-1}$ across the N=25 dataset. The reduced scattering coefficient for scalp, skull, and brain for the simulation in Fig. 2 was set to the median fitted value for the dataset, $\mu_{s}^{\prime }=0.9\;{\mathrm{mm}}^{-1}$. All other optical properties and layer thicknesses were the median values for an adult forehead given in Table 1.

**Fig. 4:**
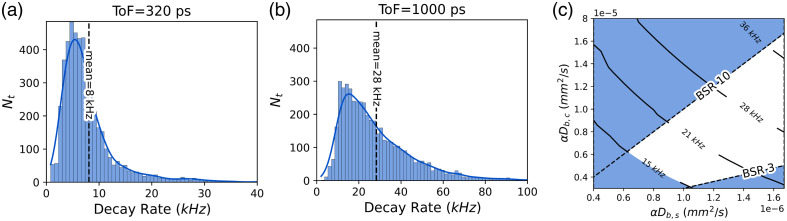
Distribution of decay rates assuming a homogeneous TD-DCS model and $\mu_{s}^{\prime }=0.9\;{\mathrm{mm}}^{-1}$ at (a) ToF = 320 ps and (b) ToF = 1000 ps measured in N=25 healthy adults. Here, $N_{t}$ represents the number of time points in a given decay rate bin across the 25 subjects for the first 200 s of recording at rest. (c) Contour plot of $g_{1}$ decay rate in simulation at 1 ns as a function of simulated $\alpha D_{B,\text{brain}}$ (CBF) and $\alpha D_{B,\text{scalp}}$ (SBF). The unshaded region indicates the range of $\alpha D_{B,\text{brain}}$ and $\alpha D_{B,\text{scalp}}$ that can explain the interquartile range of the decay rate distributions at 1 ns seen *in vivo* in panels (a) and (b). Dashed lines indicating the physiological expected bounds of brain-to-scalp flow ratio (BSR) 3–10 are also displayed.

To determine a suitable range of $\alpha D_{B}$ values to apply in the remainder of our simulations, the distribution of $g_{1}$ decorrelation rates at early and late ToFs across the N=25 dataset were analyzed. It was initially observed that, in many subjects, the experimentally measured $g_{1}s$ decorrelated faster than simulation when using typical literature values for $\mu_{s}^{\prime }$ and $\alpha D_{B}$ at 1064 nm ($\mu_{s}^{\prime }=0.84\;{\mathrm{mm}}^{-1}$, $\alpha D_{B,\text{scalp}}=1\times 10^{-6}\;{\mathrm{mm}}^{2}\;s^{-1}$). Figure 4(a) shows the measured $g_{1}$ decorrelation rate distribution at an early ToF for subjects at rest. The distribution is skewed with a large, high decay rate tail representing the decorrelation rates observed at the systolic peak.

To establish the appropriate ranges for simulated baseline $\alpha D_{B,\text{scalp}}$ and $\alpha D_{B,\text{brain}}$ that match experiment, a two-step process was employed:

1. **Examining the early ToF decay rates to fix the** ${\alpha D}_{B,\mathrm{scalp}}$ **range:** The interquartile range of decorrelation rates at early ToF [Fig. 3(a)] was used to define the range of simulated baseline $\alpha D_{B,\text{scalp}}$ for this study as $(0.5\times 10^{-6}\text{ to }1.7\times 10^{-6})\;{\mathrm{mm}}^{2}\;s^{-1}$, where $\alpha D_{B,\text{scalp}}$ is recovered from the gradient of the early ToF decorrelation rate, as per Eq. (8).
2. **Examining the late ToF decay rate distributions to fix the** $\alpha D_{B,\text{brain}}$ **range:** The determination of an appropriate range for $\alpha D_{B,\text{brain}}$ is more involved. The added complexity is due to the dependence of measured decorrelation rates on individual subject’s tissue layer thicknesses and optical properties coupled with the unavailability of distance-to-brain measurements for all 25 subjects. To address this, scalp/skull thicknesses within one standard deviation of the population mean (3 to 9 mm skull thicknesses, representing the middle 68% of the population) were simulated. This simulation, combined with the fitted reduced scattering coefficients and the $\alpha D_{B,\text{scalp}}$ range established in step 1, allowed for the identification of the range of $\alpha D_{B,\text{scalp}}$, $\mu_{s}^{\prime }$, and $\alpha D_{B,\text{brain}}$ that could explain the inter-quartile range of the decorrelation rate distribution observed *in vivo* at ToF = 1 ns [Fig. 4(b)]. The unshaded region in Fig. 4(c) displays the identified range of $\alpha D_{B,\text{brain}}$ and $\alpha D_{B,\text{scalp}}$ values for the median case (∼12  mm distance–to–brain, 6 mm skull thickness, and mean fitted reduced scattering coefficient, $\mu_{s}^{\prime }=0.9\;{\mathrm{mm}}^{-1}$), which yielded decorrelation rates consistent with the *in vivo* data. The x-axis limits in Fig. 3(c) bound the range of $\alpha D_{B,\text{scalp}}$ that is consistent with the early ToF decorrelation rates. Meanwhile, the dashed diagonal lines indicate values where the brain-to-scalp flow ratio (BSR) falls inside the expected physiological range of BSR=3–10. This analysis facilitated the identification of an appropriate range of $\alpha D_{B,\text{brain}}$ values, which for the median distance-to-brain case are presented in Table 1.

### 3.3 Effects of ToF, SDS, and Acquisition Frequency on Brain Sensitivity in the Multilayer Slab Model

**Fig. 5:**
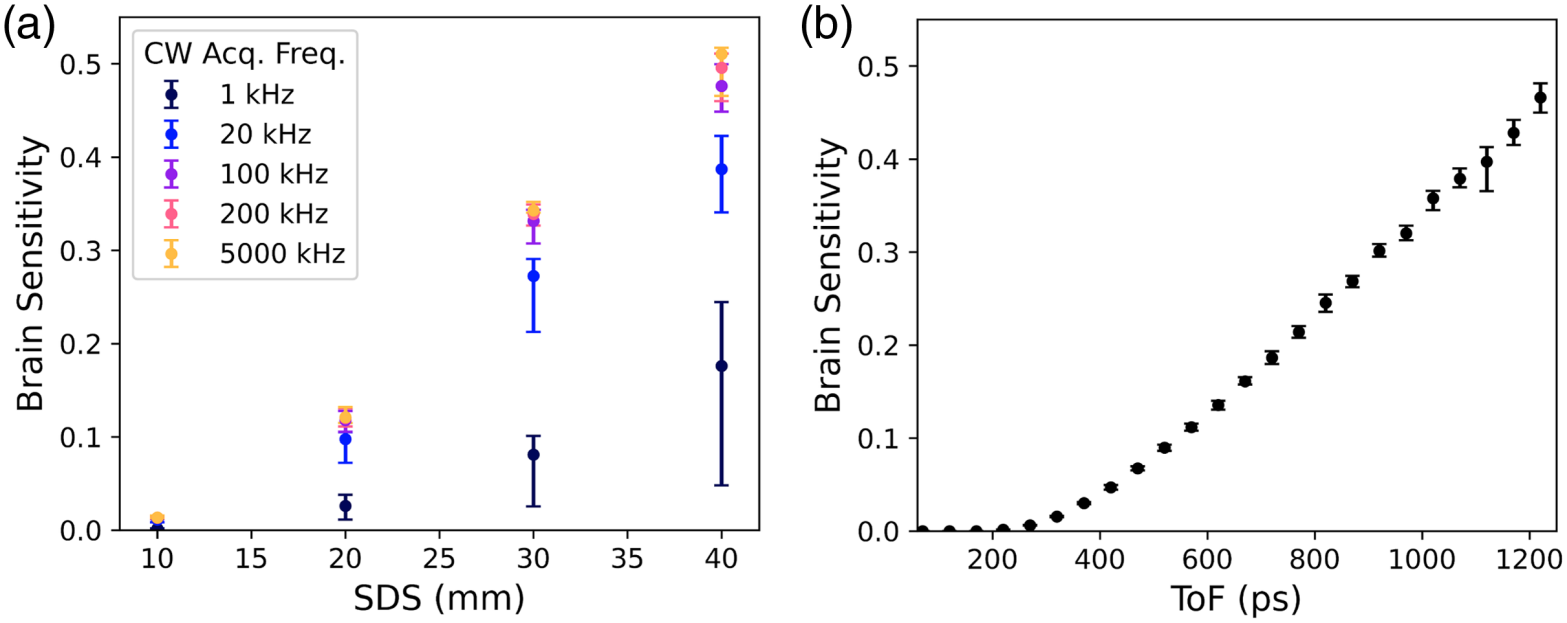
(a) Brain sensitivity (lag integrated) as a function of SDS and CW acquisition frequency for the median brain depth and default parameters given in Table 1. (b) Brain sensitivity as a function of ToF for a time-resolved, 10 mm SDS measurement with an acquisition frequency of 200 kHz.

This section evaluates the brain sensitivity of different ToF gates, source-detector separations and acquisition rate configurations in a planar simulation with scalp, skull, CSF and brain layers. The expected variances of $\alpha D_{B,\text{brain}}$ and $\alpha D_{B,\text{scalp}}$, as determined in Sec. 3.2, are included to provide error bars on the results. Figure 5(a) displays the brain sensitivity, integrated over all accessible lags, for a CW-DCS measurement as a function of SDS at different acquisition frequencies. The figure also displays the standard deviation of the distribution of brain sensitivity values obtained over all simulated values of baseline $\alpha D_{B,\text{scalp}}$ and $\alpha D_{B,\text{brain}}$. Brain sensitivity is strongly dependent on both SDS and acquisition frequency. In the median (default) parameter case, a brain sensitivity of ∼0.45 is achieved at 40 mm SDS for the highest acquisition frequency of 5000 kHz. Figure 4(b) displays an equivalent plot for a time-resolved measurement with an acquisition frequency of 200 kHz and and SDS of 10 mm (the specifications of CoMind R1). Brain sensitivity increases approximately linearly with ToF, and a brain sensitivity of ∼0.45 is achieved at a ToF of 1.2 ns in this median (default) case. Optical properties and layer thicknesses are the same for the simulations in both Figs. 5(a) and 5(b).

**Fig. 6:**
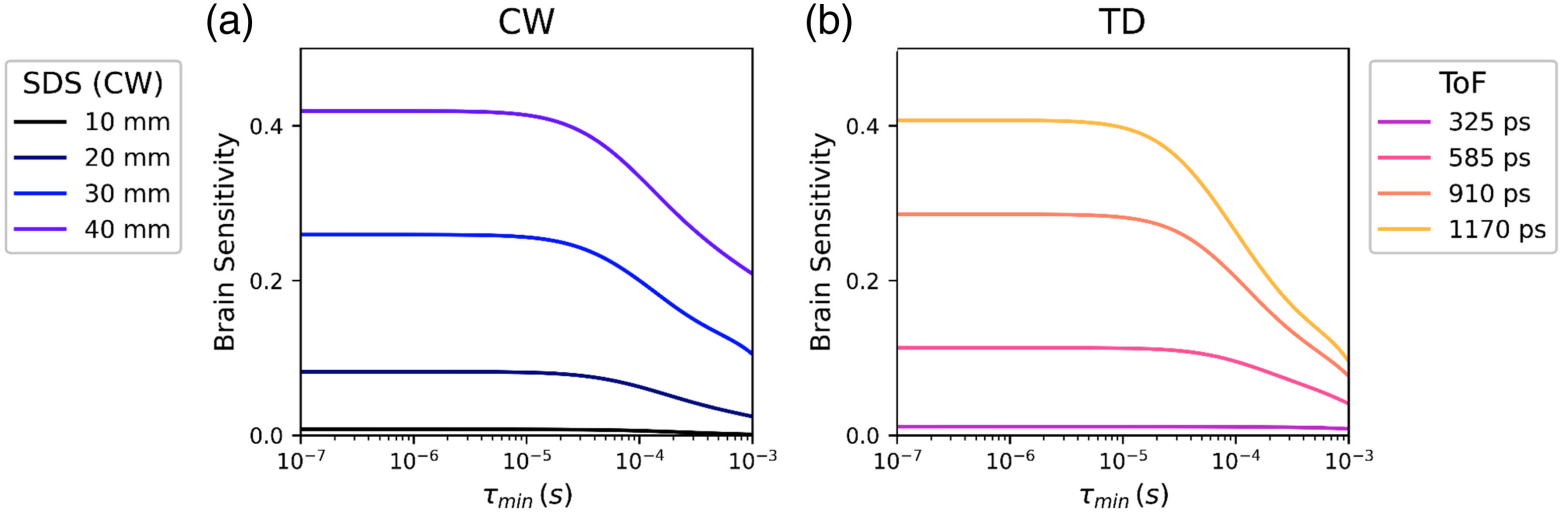
(a) Brain sensitivity (lag integrated) as a function of the minimum resolvable lag for CW-DCS at a range of SDS. (b) Brain sensitivity as a function of the minimum resolvable lag for a time-resolved device as determined at various ToF. Note the significant decrease in achievable brain sensitivity as minimum lag increases (i.e., as acquisition frequency decreases).

Figure 6(a) displays the brain sensitivity, integrated over all accessible lags (i.e., from the minimum lag to infinity) as a function of minimum lag for different SDS for CW measurements. Figure 6(b) shows the equivalent plot for the time-resolved case, wherein brain sensitivity is displayed as a function of minimum lag for a 10 mm SDS and a range of ToFs. In both bases, brain sensitivity is low when the minimum lag is large, but increases as lag decreases (i.e., brain sensitivity increases as acquisition frequency increases). Brain sensitivity then plateaus at lags lower than ∼(1 to 10)  μs (i.e., acquisition frequencies of 100 kHz to 1 MHz). Utilizing acquisition frequencies slower than this can therefore dramatically limit brain sensitivity, even for long SDS or late ToFs.

**Fig. 7:**
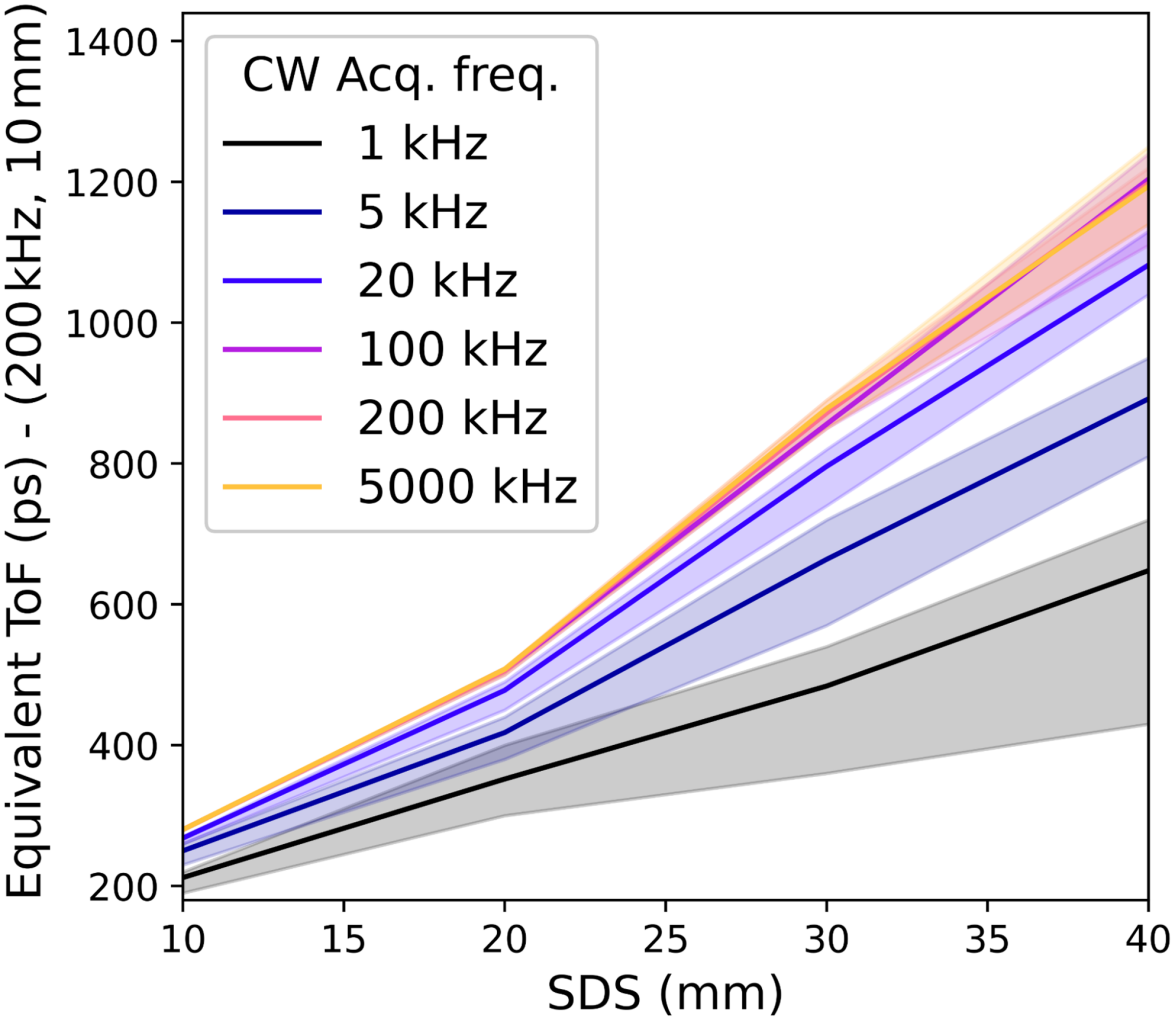
ToF required for a 200-kHz time-resolved measurement with a 10-mm SDS to achieve the equivalent (lag integrated) brain sensitivity as a CW-DCS measurement with the given acquisition frequency and SDS. A time-resolved measurement with these specifications that can achieve a ToF of >1  ns will outperform CW devices with SDS≤35  mm, whereas a ToF of 1.2 ns is equivalent in brain sensitivity to a CW measurement at an SDS of 40 mm.

Figure 7 provides a direct comparison of CW and ToF-resolved cases. It depicts the ToF at which the brain sensitivity of a time-resolved measurement (at 10 mm SDS and with an acquisition frequency of 200 kHz) matches that of a CW-DCS measurement with the stated SDS and acquisition frequency. Again, the shading represents the standard deviation associated with the range of simulated $\alpha D_{B,\text{brain}}$ and $\alpha D_{B,\text{scalp}}$ values provided in Table 1. At 10 mm SDS and 1.2 ns ToF, a time-resolved measurement has approximately equivalent brain sensitivity to a 40 mm SDS CW-DCS measurement with the same or higher acquisition frequency.

### 3.4 Simulation of Spatial Distributions of Sensitivity in an Anatomical Head Model

**Fig. 8:**
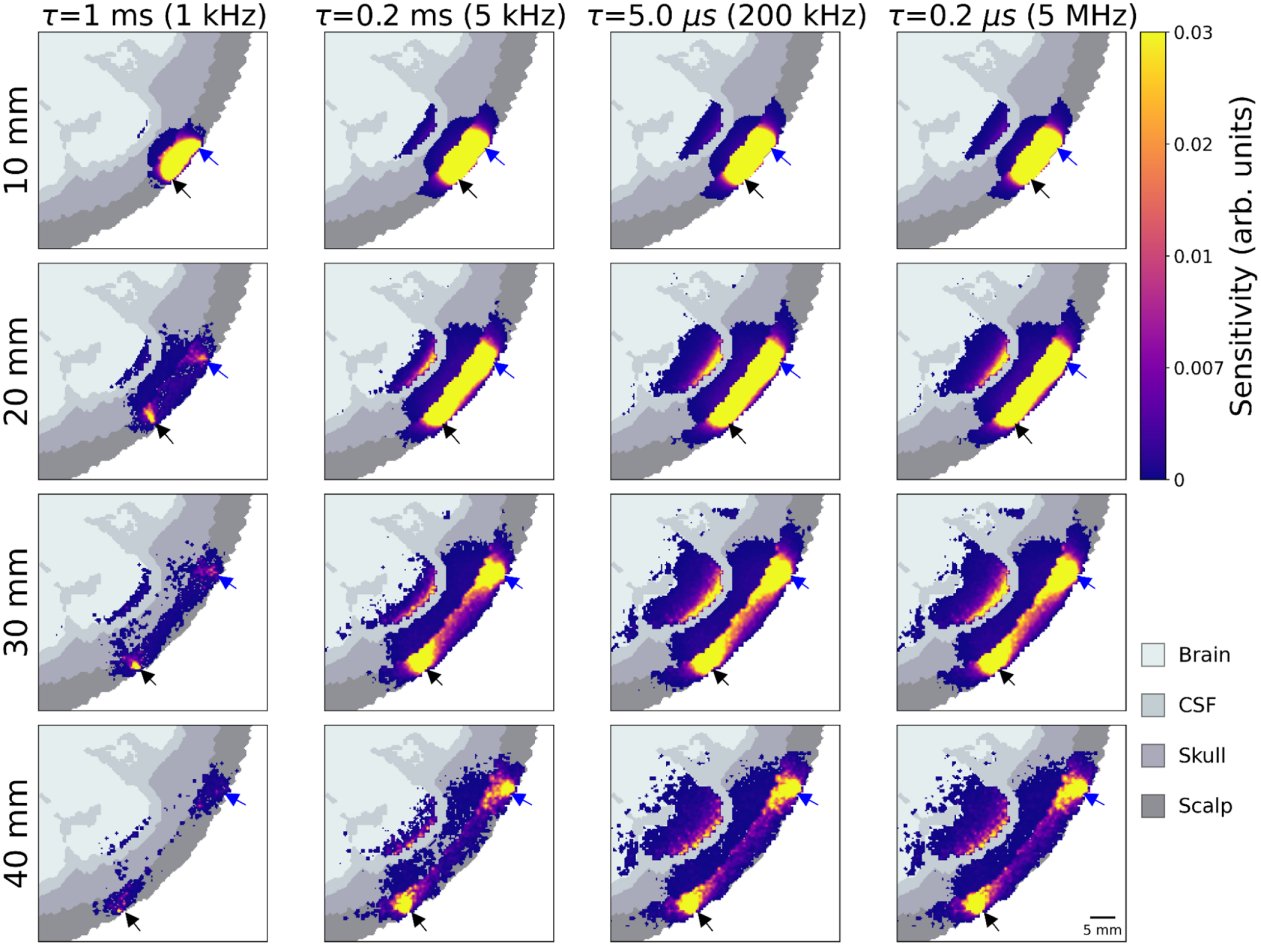
Spatial sensitivity maps at a given SDS (rows) for different minimum lags (columns) for a CW-DCS device.

Figure 8 displays a matrix of heat maps of sensitivity integrated from some minimum lag to relative changes in $\alpha D_{B}$ for different ToFs and acquisition frequencies in the anatomical head simulation. As expected, the sensitivity at 10 mm SDS is almost entirely scalp-dominated for all acquisition frequencies. At 20 mm, some sensitivity to the brain layer is evident, although it is noticeably smaller for 5 and 1 kHz acquisition frequencies. This pattern continues for longer SDS: brain sensitivity increases with SDS but only if the acquisition frequency is sufficient. At a 5 kHz acquisition rate (a 200-μs sample time), even at long SDS, the sensitivity to the brain remains limited.

**Fig. 9:**
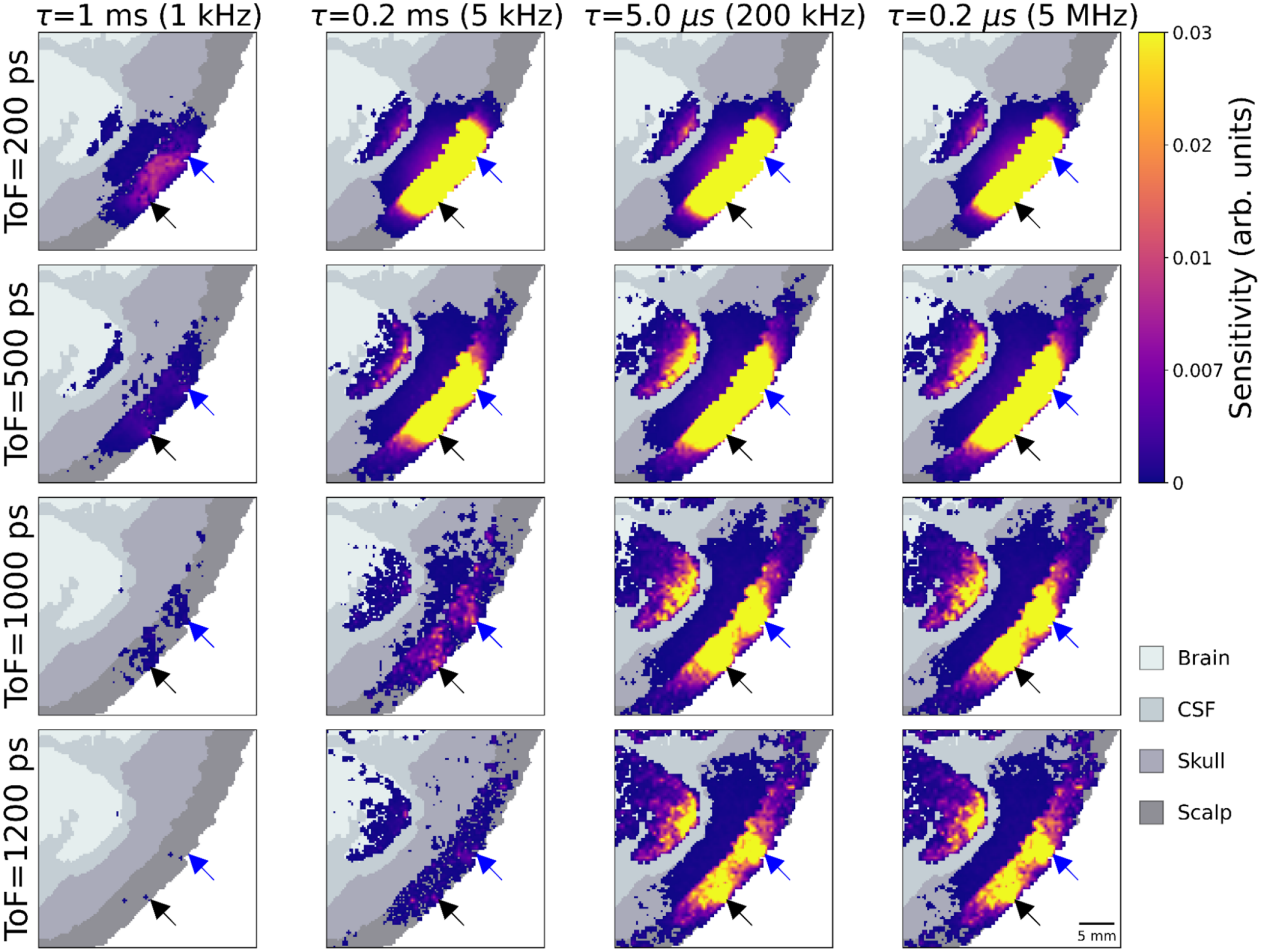
Spatial sensitivity maps at a given ToF (rows) for different minimum lags (columns), for a time-resolved measurement with a 10-mm SDS.

Figure 9 presents the equivalent figure but for time-resolved measurements at a range of ToF but a single SDS (10 mm). An analogous pattern is evident to that of Fig. 8; here increasing ToF increases sensitivity in the brain layer, but only when the acquisition rate is sufficiently high.

### 3.5 Recovery of Pulsatile BFi

**Fig. 10:**
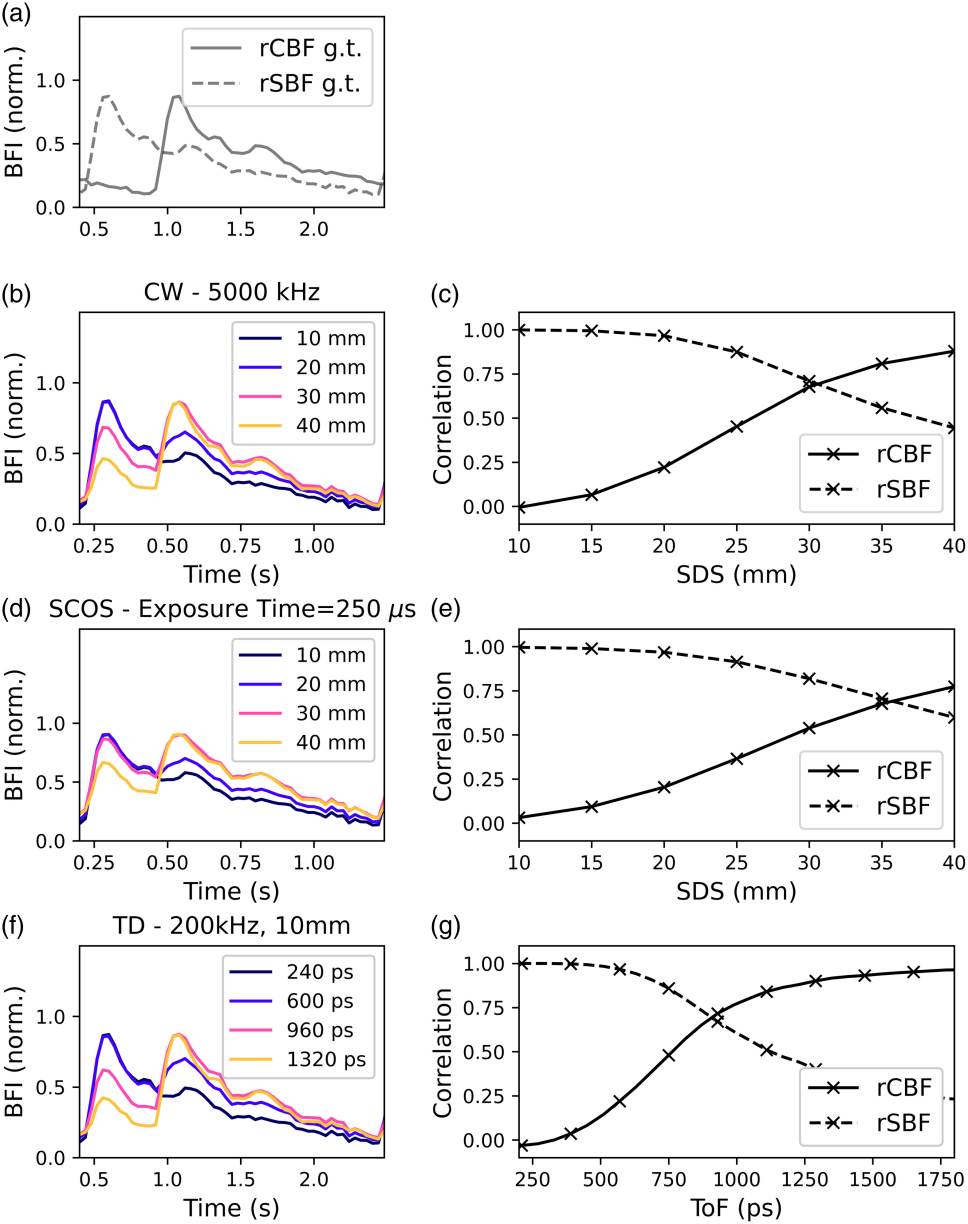
(a) Ground truth rCBF and rSBF waveforms. (b) A snippet of the rBFi time series recovered for the CW-DCS case for different SDS, assuming a 5000 kHz acquisition frequency. (c) Correlation of this recovered rBFi with the ground truth cerebral and scalp blood flow signals as a function of SDS. (d) The recovered rBFi from a SCOS-like measurement with a 250 μs exposure time and (e) the correlation of the recovered rBFi with the ground truth CBF/SBF as a function of source-detector separation for this SCOS case. (f) The rBFi time-series snippet for a time-resolved measurement at a range of ToFs, and (g) the correlation between those recovered rBFi waveforms and rCBF and rSBF waveforms as a function of ToF. Each result is based on the median adult brain depth and optical properties presented in Table 1. Note the crossing points in figures (c), (e), and (g).

In this section, we investigate the recovery of pulsatile BFi, a typical clinical neuromonitoring target, for the median optical properties and layer thicknesses outlined in Table 1, the methods for simulating a pulsatile time series are provided in Secs. 2.6 and 2.7. Figure 10 presents the simulated (ground truth) and recovered pulsatile BFi waveforms for a simulated CW-DCS-like device with a 5000-kHz acquisition frequency, a simulated CW-SCOS-like device with an exposure time of 250  μs, and a simulated time-resolved device with a 10 mm SDS and a 200 kHz acquisition frequency. Figure 10(a) shows the ground truth simulated rCBF ($\alpha D_{B,\text{brain}}$) and rSBF ($\alpha D_{B,\text{scalp}}$) waveforms. Figure 10(b) shows the recovered rBFi pulse waveform at four different SDS for a CW-DCS device, whereas Fig. 10(c) displays the correlation of those rBFi waveforms with the ground truth rCBF (solid) and rSBF (dashed) lines. As one would expect, the correlation between the recovered waveform and the rCBF waveform increases with SDS, whereas the correlation with the rSBF waveform decreases. The corresponding plots for the SCOS simulation results are presented in Figs. 10(d) and 10(e). For the CW-DCS measurement, the correlation with rCBF and rSBF crosses over at an SDS of ∼31  mm, for SCOS the cross over occurs at 35 mm. When the SDS exceeds this “crossing SDS,” the recovered BFi pulse waveform is more representative of pulsatile blood flow in the brain layer than it is of the scalp layer for this median brain-depth model. At SDS <31  mm, the recovered BFi waveform is dominated by pulsatile flow in the superficial layer.

Figures 10(f) and 10(g) present the equivalent results for the time-resolved measurement. Figure 10(f) shows the recovered BFi waveforms for a range of ToFs, whereas Fig. 10(g) shows the correlation with the simulated rCBF and rSBF waveforms as a function of ToF and provides a “crossing ToF” of 0.9 ns, after which the recovered BFi is more representative of the brain than it is of the scalp for this median brain-depth simulation.

Note that a crossing SDS of 31 mm and crossing ToF of 0.9 ns, is consistent with the result presented in Fig. 7, which was derived based purely on the fundamental sensitivity to changes in $\alpha D_{B}$ rather than on the ability to explicitly recover a pulsatile CBF waveform.

The position of the crossing ToF and crossing SDS were found to be largely insensitive to the relative phase shift between the pulse waveform in the scalp and brain layers and to the morphology of the waveform itself (for a given acquisition rate and measurement modality). Changing the ground-truth waveform (for fixed mean optical properties and pulsatility index) tends to change the absolute correlation of recovered rBFi to both CBF and SBF in a similar manner. The ToF or SDS at which the two correlations “cross-over” thereby remain relatively unaffected by waveform morphology. These results are presented in more detail in the Supplementary Material.

The key independent variables that do significantly affect these crossing points are the chosen baseline optical properties, the brain-to-scalp flow ratio, and (most critically) the layer thicknesses. An increase in IRF width for the time-resolved case will also tend to push the crossing point out to a later ToF (see Supplementary Material).

### 3.6 The Effect of Brain Depth on Brain Sensitivity

Thus far, the results presented have focused on simulations with layer thicknesses approximating the median adult forehead (4 mm scalp, 6 mm skull, and 2 mm CSF). However, any clinically relevant device must be capable of capturing a very high proportion of the full distribution of brain depths in the target population. In Sec. 3.5, crossing ToF and crossing SDS points were identified, where the recovered rBFI shows equal correlation to the brain and scalp signals. These points were found to be largely independent of waveform morphology. In this section, we expand on this analysis by investigating how the distance to the brain affects the positions of these crossing points.

**Fig. 11:**
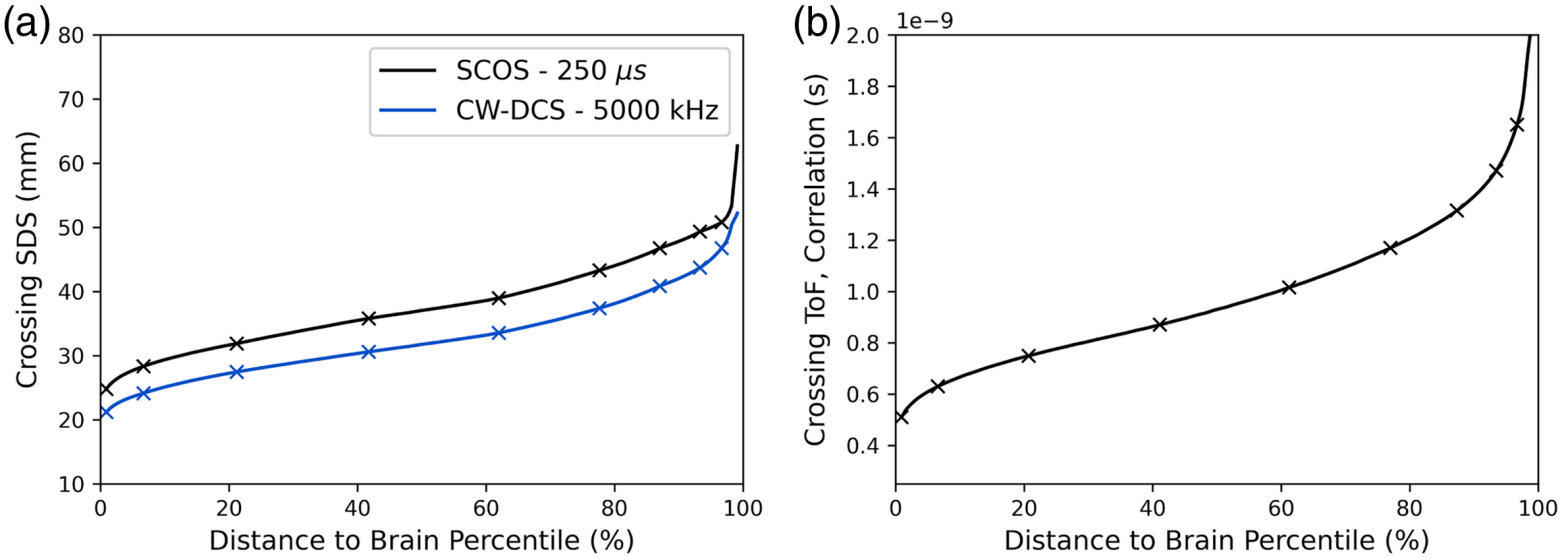
(a) Crossing SDS (for simulated CW-DCS and SCOS measurements) as a function of distance to brain population percentile. (b) The crossing ToF as a function of distance-to-brain population percentile for a time-resolved measurement.

Figure 11(a) shows the estimated crossing SDS for a simulated CW-DCS measurement (at 5000 kHz acquisition frequency) and a representative SCOS measurement (at 250  μs exposure time) as a function of the distance-to-brain population percentile. The crossing SDS and crossing ToFs were determined for brain depth values from 9 to 18 mm in 1 mm increments (crosses in Fig. 11), spanning the observed anatomical range on the forehead.^36^^,^^52^ Note that the 50th and 98th percentile distance-to-brain for the forehead are 12.4 and 17.8 mm, respectively, with almost all the variance (to the nearest mm) explained by changes in skull thickness (see Supplementary Material). Figure 11(b) displays the crossing ToF versus distance-to-brain population percentile, as recovered from simulated time-resolved measurements with an SDS = 10 mm and a 200-kHz acquisition frequency.

The results of Fig. 11(a) suggest that to measure a pulsatile BFi signal that is more representative of the brain than the scalp in greater than 85% of the adult population requires an SDS of at least 40 mm for the simulated CW-DCS case, and at least 46 mm for the SCOS case, when both techniques are operating at 1064 nm, in the noise-free limit. To achieve the same goal in 95% of the adult population requires an SDS of at least 45 mm for CW-DCS and at least 50 mm for SCOS.

The results of Fig. 11(b) suggest that for a time-resolve measurement, a ToF gate of over 1.2 ns would provide an rBFi signal that is more representative of the brain than the scalp in 85% of the adult population, whereas a ToF of 1.5 ns can achieve the same target in 95% of the adult population.

## 4 Discussion

SCOS and CW-DCS have been compared in experiments and simulations. Majeski et al.^67^ showed, in armcuff measurements, how the temporal and lag sampling characteristics of the different technologies can influence the recovered BFi pulse shape. Meanwhile, Robinson et al.^16^ demonstrated, in simulations, how the brain sensitivity of SCOS devices decreased with increased exposure time, mirroring the decline in brain sensitivity with increasing minimum resolvable lag observed in this study. Comparisons between TD-DCS and CW-DCS have been conducted by Cheng et al.^40^ and Huang et al.^41^ These comparisons are specific to TD-DCS technologies based on pulsed laser-sources with limited ToF resolution, broad IRFs (>300  ps) and short coherence lengths. Under these conditions the benefits of TD measurements are inherently limited. However, the development of interferometric measurement methods have enabled time-resolved measurements with narrow IRFs, small ToF bin sizes, and long coherence lengths.^44^^,^^45^ This paper aims to provide a more thorough investigation into the relative benefits of ToF gating, SDS gating, and lag gating when the temporal sampling is not a limiting factor.

Herein, we have described an extensive series of Monte Carlo simulations of ToF resolved measurements of CBF. Simulations were validated against both phantom and *in vivo* measurements. The *in vivo* validation allowed the direct calibration of the variance in $g_{1}$ decay estimates and optical properties from a relevant population, whereas comparison with phantom measurements provided an explicit demonstration of the consistency of our simulated time-resolved measurements with experimental data when optical properties were known. Agreement between phantom and simulations was good for TPSFs and $g_{1}$ curves for the bilayer cases measured (intralipid concentrations with a $\mu_{s}^{\prime }$ of $0.55\;{\mathrm{mm}}^{-1}$ in top layer and 0.55 to $2.6\;{\mathrm{mm}}^{-1}$ in the bottom layer). Our simulated results for ξ versus ToF [Fig. 2(e)] demonstrate, in general, excellent agreement with both homogeneous and bilayer phantom data. However, at late ToFs, those exceeding 1.2 ns, the experimental ξ deviates from its expected linear trend [Fig. 2(e)] due to declining signal-to-noise ratio. Furthermore, a minor offset between simulated and experimental ξ is observed for the homogeneous case, which is hypothesized to result from a slight misalignment between the simulation and experimental ToF zero. Note that the agreement between experimental and simulated $g_{1}s$ is worse at early ToFs, <200  ps (Fig. 2). Possible explanations for this discrepancy are a mismatch between simulated and experimental IRFs and/or a breakdown of the assumption that scatterer motion is well modelled as Brownian at very early ToFs. The range of simulated $\alpha D_{B,\text{scalp}}$ and $\alpha D_{B,\text{brain}}$ that matched *in vivo* measurements are consistent with typical values measured in the literature,^37^^,^^38^^,^^54^^,^^56^ although it was found that the mean values of the distribution of $\alpha D_{B,\text{scalp}}$ and $\alpha D_{B,\text{brain}}$ were slightly higher and lower, respectively, than those used in previous simulation studies ($\alpha D_{B,\text{scalp}}=10^{-6}\;{\mathrm{mm}}^{-2}$ and $\alpha D_{B,\text{brain}}=6\times 10^{-6}\;{\mathrm{mm}}^{-2}\;s^{-1}$).^36^^,^^48^ Baseline $\alpha D_{B,\text{scalp}}$ during the occluded state is consistent with a medium with only a very small number of dynamic scatterers. The fitted reduced scattering coefficient for the 25 subjects, $\mu_{s}^{\prime }=(0.9\pm 0.3)\;{\mathrm{mm}}^{-1}$, is consistent with the values at 1064 nm compiled by Jacques.^68^

Using this simulation stack, the effects of acquisition frequency, ToF, and source-detector separation on various brain sensitivity metrics were investigated. It is important to highlight that this simulation stack neglects the effects of hardware noise. In CW-DCS, noise is typically non-uniform across autocorrelation lags,^69^^,^^70^ with an increased noise contribution at shorter lags. Noise effects are more significant at higher sampling rates and faster autocorrelation decays. Noise considerations are particularly important when comparing the theoretical performance of SCOS with lag-resolved methods. SCOS can achieve higher detected photon counts through parallel detection across camera pixels but is affected by dark, shot, and readout noise from the sensor.^71^ These contributions increase with decreasing exposure time.^72^ Because these noise components are integrated over the entire exposure, they disproportionately affect the SNR of the rapid, lower-amplitude speckle fluctuations associated with deeper photon paths, thereby reducing brain sensitivity. Comparing different measurement approaches and device parameters in the noise-free limit must therefore be done with caution. The results presented here provide insights into the theoretically achievable performances of different measurement approaches, and the implied performance targets might best be considered to represent lower bounds on the required performance of any real-world instrument.

The increase in noise observed at higher sampling rates and for faster decorrelation regimes arises naturally from the limited photon budget available per sampling interval. Under such conditions, photon shot noise becomes dominant, particularly at longer ToFs where photon flux is lower.^44^ This trend aligns with diffusing wave spectroscopy (DWS), in which the decay rate of the field autocorrelation $g_{1}$ scales predictably with ToF, reduced scattering coefficient, and wavelength. Thus, the ToF-dependent behavior of the measured signal-to-noise ratio supports our recommendation to balance photon statistics and flow sensitivity when selecting the optimal measurement window. Practically, these noise effects can be mitigated by increasing the number of parallel detection channels or applying adaptive integration strategies that extend averaging time at longer ToFs. These measures enhance the effective SNR without compromising temporal resolution. Although not reported here, we have also developed a comprehensive end-to-end simulation of the iNIRS system, encompassing photon propagation, interferometric detection, and statistical noise modeling. This framework enables systematic optimization of acquisition parameters and quantitative prediction of performance trade-offs, but its detailed presentation is beyond the scope of the current manuscript.

The impact of speckle coherence and sampling statistics are also not modelled here. In reality, non-interferometric approaches such as CW-DCS, TD-DCS, and SCOS, which are sensitive only to light intensity and not phase, will incur additional signal degradation when translating from $g_{2}$ to $g_{1}$ via the Siegert relation.^73^ This translation introduces a bias into the measured decay rates, with errors becoming larger when speckle coherence is low (when β is small).^74^^,^^75^ The results presented here do not account for these effects and therefore likely overestimate the theoretical performance of non-interferometric approaches.

A key result of this simulation work is the identification of a “brain sensitivity cliff” (Fig. 6), wherein the sensitivity to changes in CBF rapidly decreases for a minimum lag greater than 10  μs (i.e., acquisition rates below 100 kHz) for both CW and TD devices. This same effect can be seen in the spatial sensitivity maps of Figs. 8 and 9. Even at late ToFs or long SDS, if the minimum lag is ∼1  ms, there is almost no brain sensitivity. However, it is important to re-iterate that all the simulations presented here are based on a wavelength of 1064 nm. Because of the higher $\mu_{s}^{\prime }$ values associated with more traditional NIRS wavelengths, this “brain sensitivity cliff” almost certainly occurs at much shorter minimum lags for wavelengths in the 700 to 800 nm range. This will undoubtedly result in higher requirements on acquisition frequency at those wavelengths.

A downstream impact of the depth sensitivity fall-off for τ>10  μs is that only relatively minor *theoretical* brain sensitivity improvements are predicted by increasing acquisition frequencies beyond 100 kHz. This is consistent with findings by Selb et al. and Robinson et al.^16^^,^^28^ For the practical recovery of pulsatile BFi, however, it is important to also take into account the fitting of the $g_{1}/g_{2}$ curves and the robustness of this procedure to noise.

In Fig. 7, the theoretical brain sensitivity of CW-DCS at different SDS is compared with a simulated time domain measurement with a 10-mm SDS and a 200-kHz acquisition rate. Figure 7 demonstrates that a time-resolved measurement at a ToF of ∼0.9  ns provides equivalent brain sensitivity to a typical CW-DCS measurement at an SDS of ∼30  mm. At a ToF of 1.2 ns, a time-resolved measurement will be approximately equivalent to a CW-DCS measurement at an SDS of 40 mm.

To explicitly investigate the recovery of pulsatile CBF waveforms, we also simulated multi-layer, pulsatile changes in $\alpha D_{B}$ and examined the recovered pulsatile rBFi time series for time-resolved, CW-DCS and SCOS cases. We found that a waveform-invariant metric for assessing the brain sensitivity of a given modality is the ToF or SDS at which the correlations of the recovered BFi with CBF and with SBF intersect. These crossing points represent the ToF/SDS beyond which the recovered BFi is more representative of brain flow than it is of scalp flow. For the default (median) brain depth, these crossing points occur at 31 mm SDS for CW-DCS, 35 mm SDS for SCOS, and 0.9 ns ToF for the simulated time-resolved measurement.

The “crossing SDS” and “crossing ToF” were also examined as a function of distance-to-brain, rather than just the median case, as any successful neuromonitoring device must be applicable to the vast majority of the target population. The results of this analysis (Fig. 11) show that to achieve a BFi signal that is more representative of the brain than the scalp in 85% of the population requires an SDS of ∼40  mm for CW-DCS and ∼46  mm for SCOS, or a ToF of ∼1.2  ns for time-resolved measurements, assuming operation at 1064 nm. To be effective, it is of course necessary to achieve these targets while also recovering BFi with high SNR and at sample rates sufficient to resolve the pulsatile signal. The state of the art of CW-DCS and SCOS (and related methodologies such as interferometric diffusing wave spectroscopy) are now approaching, or in some cases exceeding, this level of performance.^49^^,^^76^^,^^77^ However, to-date no time-resolved methodology has demonstrated the capacity to resolve pulsatile BFi at greater than ∼700  ps.^19^^,^^78^

Although these “crossing points” represent an arbitrary target of equal contributions from the scalp and brain layers, achieving such parity is a high bar in tissue optics terms. CW-NIRS measurements in adults very rarely achieve a raw signal that is more representative of the brain than it is of the scalp.^29^ However, it is important to note that achieving this signal parity is not, on its own, sufficient for a robust, clinically meaningful measure of CBF. The recovered BFi clearly remains heavily influenced by scalp blood flow (as the full range of simulations presented here demonstrate). Additional signal processing techniques are essential to isolating a truly brain-specific CBF signal that is suitable for clinical use and this will almost certainly require independent measurements of scalp flow to permit signal de-mixing. Time-resolved methods offer a clear advantage here: by capturing measurements at multiple ToF, they provide a level of depth specificity that is impossible to replicate in the CW domain without a multi-SDS configuration.

Beyond the fundamental mechanisms of ToF gating, SDS gating, and lag gating, brain sensitivity can also be increased by optimizing the algorithms used to extract BFi from $g_{1}$ or $g_{2}$. Models have been developed that attempt to explicitly separate the brain from the superficial flow signals. Methods used to-date include reduced analytic forms,^17^ multilayer solutions to the correlation-diffusion equation,^79^^–^^81^ Monte Carlo fitting methods,^66^^,^^82^^,^^83^ and black box deep-learning-based methods.^84^ The multi-dimensional nature of time-resolved measures of BFi provide a particularly exciting opportunity to investigate and develop new, brain-selective signal processing methodologies that can complement advances in hardware to maximize brain sensitivity and selectivity.

## 5 Conclusions

Brain sensitivity has been studied extensively for CW-NIRS,^85^ CW-DCS,^28^^,^^31^ SCOS,^16^ and time-domain NIRS.^34^^,^^86^^,^^87^ Here, using a novel simulation stack, we have examined the brain sensitivity of time-resolved and CW measures of BFi at 1064 nm in the noise-free limit. We also quantified the relative advantages of ToF gating, SDS gating, and lag gating for the first time.

We found that increasing the minimum lag beyond ∼10  μs (i.e., reducing acquisition frequency below ∼100  kHz) results in a marked fall-off in brain sensitivity, even for long SDS and long photon times of flight. The relationship between ToF gating for a TD device and SDS gating for a CW device was also examined. We found that a ToF of 1.2 ns provides approximately equivalent brain sensitivity as a CW-DCS measurement with an SDS of 40 mm at 1064 nm.

By simulating multi-layer pulsatile flow, we also identify the SDS and ToF thresholds (or “crossing points”) at which the recovered BFi is more representative of the brain layer pulse than it is of the scalp. For a typical adult brain depth, this occurs at an SDS of 31 mm for CW-DCS methods, an SDS of 35 mm for SCOS, and a ToF of ∼0.9  ns for time-resolved methods. However, by examining the relationship between these crossing points and brain depth, we show that to achieve the same sensitivity threshold for 85% of the adult population requires an SDS of at least 40 mm for CW-DCS or 46 mm for SCOS, or a ToF of 1.2 ns for time-resolved methods.

Using extensive, physiologically validated Monte Carlo simulations, this work identifies key minimum device requirements that must be met to realize a clinically viable optical monitor of CBF.

## Supplementary Material

10.1117/1.JBO.13.2.025003.s01

## Data Availability

Code used to produce pulsatile simulations and a subset of figures presented in this paper is available at: https://github.com/CoMind-Technologies/mc_analysis. Data underlying the results presented in this paper are not publicly available at this time but may be obtained from the authors upon reasonable request.
