# Supplementary material for: Monte Carlo simulations of time-resolved blood flow index: times-of-flight beyond ∼1 ns are necessary for brain-dominated measurements
Source: Neurophotonics. 2026 Mar 30;13(2):025003. doi: 10.1117/1.NPh.13.2.025003 (PMC13041720; doi:10.1117/1.NPh.13.2.025003)
Supplement: Supplementary file 1 [file NPh_013_025003_SD001.pdf]

Supplementary Material

1. *Sensitivity to αD_B changes*

The sensitivity of g_1 to a change in αD_B in layer 'n', $\partial_{\alpha D_{B,n}} g_1(t, \tau_j)$, may be directly computed from a Monte Carlo simulation by computing the derivative of the expression for G_1 in Eq. 1 with respect to the value $\alpha D_{B,n}$ in layer n and normalising by the first lag. This yields the following expression,

$$\frac{\partial g_1(t_s, \tau_d)}{\partial \alpha D_{B,n}} = -\frac{1}{\sum_k^{N_p} w_k} \sum_{n=1}^{N_p} 2 Y_{n,j}(t_s) k_0^2 \tau_d \exp\left(-2 \sum_i^{N_m} Y_{n,j}(t_s) k_0^2 \alpha D_{B,n} \tau_d\right) w_j \qquad (9)$$

where, $w_j = \exp(-\sum_n^{N_m} \mu_{a,n} L_{p,n,j}(t_s))$ is the 'weight' of the j th photon the detector, N_m is the number of tissues and $L_{p,n,j}(t_s)$ is the partial path length of the j th photon detected in ToF bin t_s through the nth layer/medium. $Y_{n,j}(t_s)$ is its equivalent, but for the momentum transfer. Note Eq. 9 is equivalent to computing g_1 sensitivity with numerical differences but with the benefit of lower compute cost and improved accuracy. For the CW case the above expression remains the same but the ToF integrated g_1 should be used instead.

2. Distance-to-brain and skull thickness population distributions for the prefrontal lobe

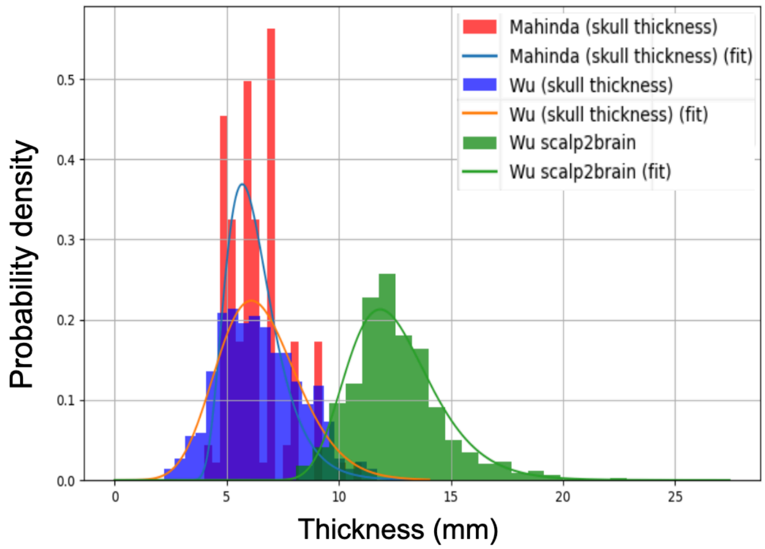

Fig. S1 Population layer thickness distributions extracted from Wu et al. and Mahinda et al. datasets[36,46]. Lines indicate fits of a log normal distribution to each data set. Note how the variance in brain depth (green) is almost identical to the variance in skull thickness (red and blue), suggesting skull thickness is the primary driver of brain depth variance in adults.

Figure S1 displays distance-to-brain and skull thickness distributions as extracted from Wu et al. and Mahinda et al. datasets [36,46]. Note that almost all the variance in brain depth (green) can be explained by the variance in skull thickness (blue and red histograms).

3. *Effect of variation in baseline optical properties on crossing ToF*

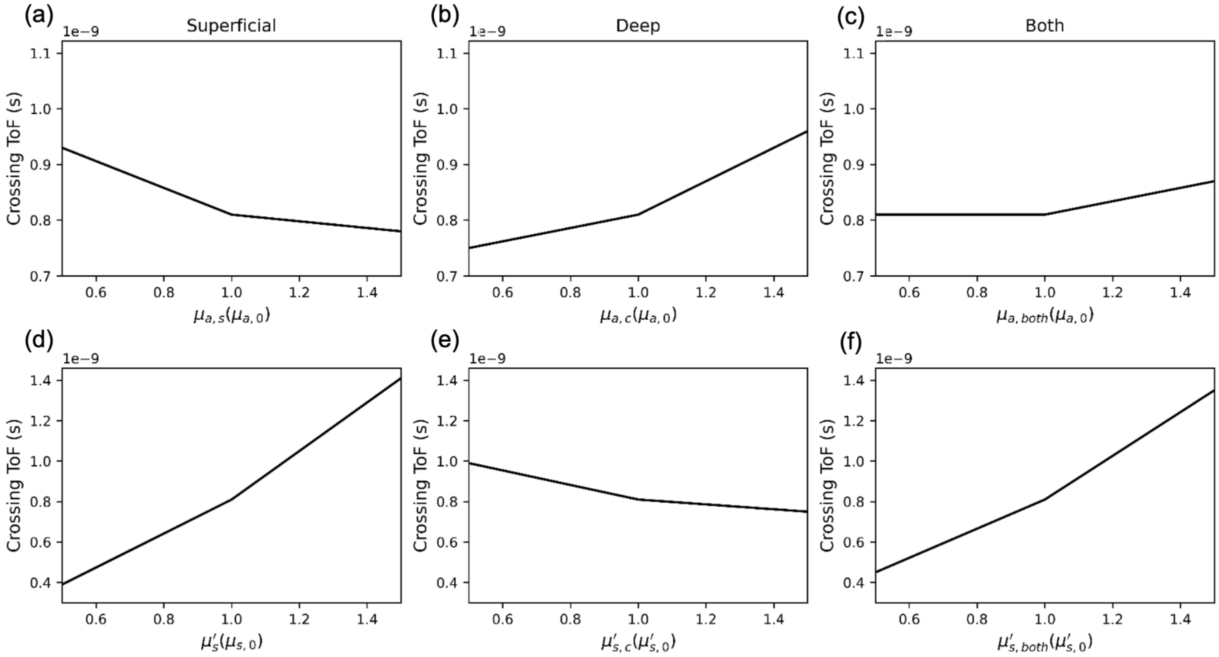

Fig. S2 From left to right, the columns show the effect of changing optical properties in superficial, deep and both layers simultaneously on the crossing ToF respectively. The upper row shows the effects of changing absorption coefficient, while the bottom row shows the effect of changing reduced scattering coefficient. All other properties are as per Table 1. X axes are normalised by the default absorption and scattering coefficients in Table 1 ($\mu_{a,0}$ and $\mu_{s,0}'$).

Figure S2 illustrates how variations in the baseline tissue absorption and reduced scattering coefficients affect the crossing ToF. Specifically, Fig.s S2 a-c) examine the impact of changing absorption in superficial, deep and both layers simultaneously. While Fig. S2d-f) present the corresponding effects for varying baseline tissue reduced scattering coefficients. Superficial layers are the scalp, skull and CSF in this simulation, while the deep layer is the brain only.

The impact of superficial and deep variance in optical properties on depth sensitivity, as measured by changes in the crossing ToF, are consistent with the work of Martelli et al. on TD-NIRS [81]. Increasing superficial absorption tends to decrease the crossing ToF (in these noise-free simulations) as photons that spend a longer time in the superficial layers are more

likely to be extinguished. This biases the detected signal towards photons that have spent a greater time in the deeper layers. The inverse occurs for increased deep layer absorption. When absorption is increased in both layers, the effect of deep layer absorption becomes dominant.

Conversely, increasing superficial reduced scattering coefficient (Fig. S2 d)) has two effects: it decreases light mean free paths in the superficial layers, which reduces light penetration depth, and increases the momentum transfer in the superficial layer (and therefore the superficial contribution to the decorrelation rate). For larger deep layer reduced scattering coefficients, the relative increase in momentum transfer in the deep layer dominates, leading to a reduced crossing ToF. When the reduced scattering coefficient is increased in both layers, the changes in the superficial layer dominate, and the crossing ToF increases. This indicates that the crossing ToF values for measurements taken in the near-infrared spectral range, where the typical tissue reduced scattering coefficient is higher, will also be correspondingly higher than those presented in this study.

4. Waveform variance and its effect on the crossing points

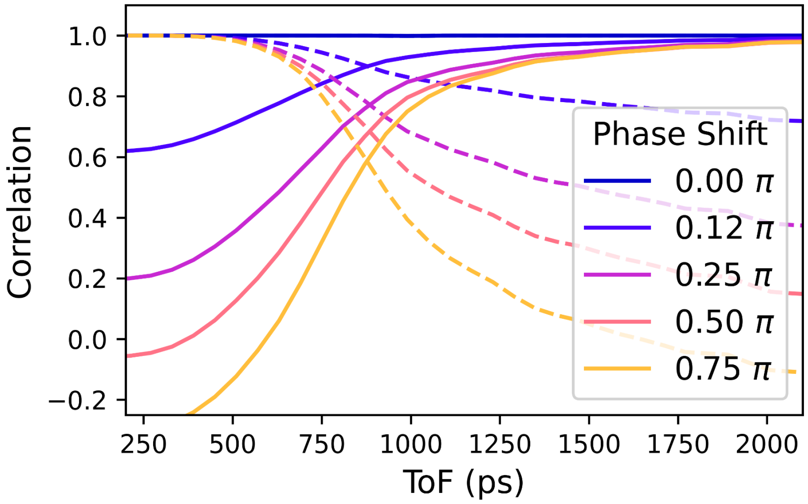

Fig. S3 Correlation of fitted BFi with simulated ground truth CBF (solid) / SBF (dashed) pulse waveforms for different phase shifts between those ground truth superficial and deep flow waveforms. As the superficial and deep flow waveforms become more distinct, the asymptotic CBF correlation values shift in magnitude while the crossing ToF is relatively unaffected.

Figure S3 shows the correlation between superficial and deep layer waveforms for different superficial and deep waveform phase shifts. As the phase shift is reduced and the waveforms become more similar, the early and late ToF CBF and SBF correlations also become more similar. In the extreme case of zero phase shift, there is perfect correlation with both scalp and brain flow at all ToF. While asymptotic early and late ToF correlation values change in magnitude with waveform phase shifts, the crossing ToF values remain almost completely fixed.

5. Effect of IRF on simulated time-resolved measurement results.

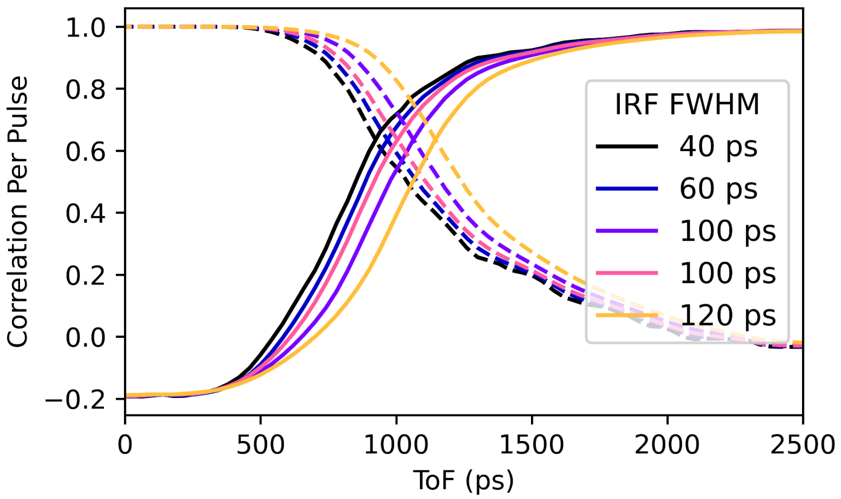

Fig. S4 Correlation between the recovered BFi time series and the ground truth CBF waveform (solid lines) and SBF waveform (dashed lines) for different simulated IRF widths.

The effect of increasing IRF width on the correlation of the recovered BFi signal to both simulated scalp and brain pulsatile blood flow waveforms is shown in Fig. S4. The IRF used in simulation, which is representative of the experimental IRF, is displayed in Fig. S5. The FWHM of this is 125 ps.

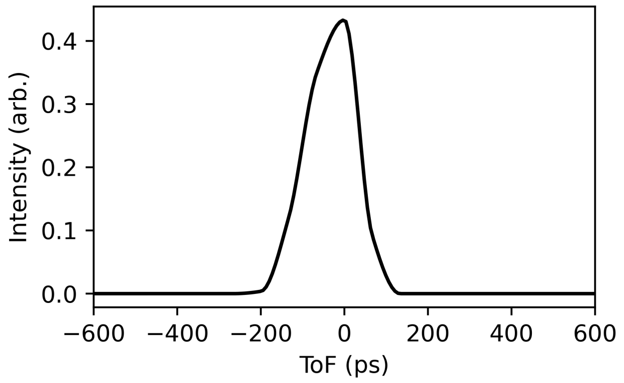

Fig. S5 Simulated IRF used in the simulation benchmarking. FWHM is 125 ps.
